# Effect of Organic Solvents on Microalgae Growth, Metabolism and Industrial Bioproduct Extraction: A Review

**DOI:** 10.3390/ijms18071429

**Published:** 2017-07-04

**Authors:** Krystian Miazek, Lukas Kratky, Radek Sulc, Tomas Jirout, Mario Aguedo, Aurore Richel, Dorothee Goffin

**Affiliations:** 1 TERRA, AgricultureIsLife, University of Liege-Gembloux Agro-Bio Tech, Passage des Déportés 2, Gembloux B-5030, Belgium; dorothee.goffin@ulg.ac.be; 2 Faculty of Mechanical Engineering, Department of Process Engineering, Czech Technical University in Prague, Technická 4, 166 07 Prague 6, Czech Republic; lukas.kratky@fs.cvut.cz (L.K.); radek.sulc@fs.cvut.cz (R.S.); tomas.jirout@fs.cvut.cz (T.J.); 3 Unit of Biological and Industrial Chemistry, University of Liege-Gembloux Agro-Bio Tech, Passage des Déportés 2, Gembloux B-5030, Belgium; mario.aguedo@ulg.ac.be (M.A.); a.richel@ulg.ac.be (A.R.)

**Keywords:** microalgae, organic solvents, cultivation, industrial effluents, extraction, industrial compounds, economic survey

## Abstract

In this review, the effect of organic solvents on microalgae cultures from molecular to industrial scale is presented. Traditional organic solvents and solvents of new generation-ionic liquids (ILs), are considered. Alterations in microalgal cell metabolism and synthesis of target products (pigments, proteins, lipids), as a result of exposure to organic solvents, are summarized. Applications of organic solvents as a carbon source for microalgal growth and production of target molecules are discussed. Possible implementation of various industrial effluents containing organic solvents into microalgal cultivation media, is evaluated. The effect of organic solvents on extraction of target compounds from microalgae is also considered. Techniques for lipid and carotenoid extraction from viable microalgal biomass (milking methods) and dead microalgal biomass (classical methods) are depicted. Moreover, the economic survey of lipid and carotenoid extraction from microalgae biomass, by means of different techniques and solvents, is conducted.

## 1. Introduction

Microalgae are photosynthetic microorganisms [1] that include cyanobacteria, green microalgae, eustigmatophytes, diatoms, dinoflagellates, coccolithophores, as well as euglenoid species, which are regarded as microalgae [2] and/or photosynthetic protists [3], and *Polytomella* species, regarded as protozoa or as unicellular colourless algae [4]. Besides photosynthetic mechanism, many microalgae strains are capable of heterotrophic and mixotrophic growth, when organic carbon sources (sugars, organic acids, alcohols, phenolics) are available [5]. Nowadays, microalgae are strongly considered as a source of lipids and carotenoids for industrial purposes [6,7]. Lipids range from fatty acids and triglycerides to phytosterols, and can be used for biodiesel production, but also as nutraceutics, food additives, antimicrobial agents and components of skin-healthcare cosmetics [6]. Carotenoids are fat soluble pigments that can find applications as food colorants, fish pigmenters and cosmetic additives. Moreover, due to their antioxidant, anti-inflammatory and anti-tumor activities, carotenoids can serve as therapeutics for the treatment of the range of health disorders, including cardiovascular diseases, cancer, skin photosensivity and eye-related diseases [7].

There is an increasing trend to produce lipids and pigments from microalgae cultivated on industrial and municipal wastewaters or landfill leachates [8,9,10,11,12]. Industrial effluents and wastewaters originate from agriculture, tannery, textile, petroleum, pulp and paper processes, pharmaceutical industry or waste landfills. However, effluents contain numerous organic and inorganic pollutants that can affect microalgae cultivation. Microalgae are susceptible to environmental pollutants such as antibiotics [13], pesticides [14] and metals [15], and besides to organic solvents [16] including ionic liquids [17], which can affect microalgal cultivation.

Organic solvents are carbon-based solvents which include methanol, ethanol, chloroform, acetone, isopropanol, butanol, ethyl acetate and hexane that differ in boiling point, density, polarity and miscibility in water. These solvents find application in food, pharmaceutical, oil and petrochemical industries [18]. Ionic liquids (ILs) are organic salts that remain in a liquid state below 100 °C. ILs possess properties such as low volatility, high solvency and non-flammability, and are considered as potential replacements for “traditional” organic solvents used in industry [19,20].

Organic solvents present in industrial effluents can have a substantial influence on microalgae cultivation. Thererefore, in this review, the effect of organic solvents on microalgae growth and metabolism is discussed to evaluate positive and negative impacts of solvents or effluents containing solvents on microalgal cultures.

On the other hand, ionic liquids and other organic solvents can be used for extraction of industrially important compounds from microalgae [21]. Notably, a great attention has been given to recovery of lipids and pigments by means of numerous extraction methods and with the use of different organic solvents [22]. In order to enable microalgal lipids and pigments to become commercialized products, high extraction yields of target products from microalge cells, have to be achieved. In this review, efficiency of lipid and pigment extraction is evaluated in terms of the type of organic solvent used and the process parameters applied. Mass and energy balances with economic survey for lipids and pigments, extracted by means of various processes (mechanical, thermal, microwave, ultrasound and supercritical fluid treatment) and different solvents (traditional solvents and ILs), were evaluated. Moreover, energy requirements and production costs for different extraction processes were calculated.

## 2. Effect of Organic Solvents on Microalgae Growth

Organic solvents possess a range of applications and can be used for extraction, synthesis, catalysis, separation, purification, cleaning, degreasing, sterilization and cryoprotection in many branches of industry [18,19,20]. Industrial usage of organic solvents can create effluents containing various organic solvents and ILs, causing potential threats to environment [23,24].

### 2.1. Industrial Wastewaters, Effluents and Streams as a Source of Solvents

Wastewaters, effluents and streams released from industry can contain numerous solvents. For instance, winery wastewaters can possess high organic content with ethanol as a major component [25], or pulp mills can emit large amount of methanol as a waste product from lignocellulose treatment [26]. Other examples come from chemical plants manufacturing and using formaldehyde, which produce wastewaters containing methanol [27] or refinery wastewaters, which are abundant sources of ethylene glycol [28]. Moreover, petrochemical wastewaters contain ethylene glycol and acetaldehyde, or BTEX (benzene, toluene, ethyl benzene, xylene) [29]. Petrochemical refineries also produce effluents containing chlorinated solvents, such as dichloromethane, chloroform, carbon tetrachloride and 1,2-dichloroethane [30]. Also, pharmaceutical wastewaters were reported to contain propanol, methanol and acetone [31], or waste organic solvents (WOS) can be generated, mainly composed of methanol, but also containing ethanol, acetone, dichloromethane, ethylacetate, tetrahydrofuran and toluene [32]. Acetone-butanol-ethanol (ABE) wastewater, originated from biobutanol production, contain residual sugars, acetic acid and butyric acid, as well as butanol and ethanol, due to the uncompleted distillation of ABE fermentation broth [33]. Beyond industry, laboratories also generate wastes containing organic solvents. For instance, chromatographic analyses create solvent wastes (mainly methanol, acetonitrile, ethanol, acetone, dichloromethane, methylethylketone) [34].

Besides traditional solvents, ILs are new generation solvents in production and in use. Even if the presence of ILs in wastewaters is not yet common, the number of applications has been increasing rapidly, which in future could lead to massive ILs releases into aqueous streams, due to waste disposal or accidental leakage [35]. Therefore, wastewaters potentially containing various ILs should not be neglected, in terms of future concerns.

Traditional solvents and ILs were reported to inhibit activity of microorganisms involved in wastewater treatment processes [36,37,38]. The presence of organic solvents in effluents can also affect microalgal cultivation and production of valuable compounds. Therefore, in the following chapters the effect of traditional solvents and ILs on microalgal growth and metabolism is depicted.

### 2.2. Effect of Traditional Organic Solvents on Microalgae Growth and Cell Metabolism

Numerous polar and non-polar organic solvents used in industry can affect microalgae growth (Table S1). Amongs them are alcohols (methanol, ethanol, propanol, isopropanol, butanol, isobutanol), ketones (acetone, butanone), amides (dimethylformamide), sulfur compounds (dimethylsulfoxide), nitriles (acetonitrile), alkanes (hexane, heptane), cycloalkanes (cyclohexane), chlorinated compounds (dichloromethane, chloroform) and aromatic compounds (benzene). These solvents enter microalgae cells via passive diffusion [39] and exert inhibitory or stimulatory effect.

#### 2.2.1. Methanol

Methanol in plants in oxidized to formaldehyde, to formate and to CO_2_, with subsequent assimilation of CO_2_ during photosynthesis [40].

Methanol at 7.9 g/L (1 *v*/*v* %) enhanced *Chlorella* sp. growth and lipid production in the presence of light and with 5% CO_2_ supplementation. Methanol assimilation was improved, when CO_2_ was supplied, and methanol addition resulted in an increase in palmitic (C16:0) and oleic (C18:1) acid content, if compared to photoautotrophic (CO_2_) control [41].

A range of methanol concentrations (0.039–0.792 g/L), (0.005–0.1 *v*/*v* %) stimulated growth of *Chlorella minutissima* within first six days of cultivation, but during the following 5 days the biomass obtained was lower than in control. The method of methanol addition, single or daily, had crucial effect on *Chlorella* growth with daily supplementation being a more favourable method [40].

Methanol at 1.6 g/L (50 mM) improved by 35% growth in *Chlamydomonas reinhardtii* culture after a few days of cultivation. During initial stage of cultivation, a 30–31% increase in protein content and free amino acid content was detected, with a change in amino acid composition: remarkably higher amounts of glutamic acid, glutamine, threonine, leucine, tyrosine and significantly lower amounts of aspartic acid, methionine, valine, histidine. An alteration of protein/amino acid profile during first cultivation hours can be due to a shift of *Chlamydomonas* cell metabolism towards methanol utilization [42].

Cultivation of *Chlorella sorokiniana* in the presence of methanol 0.5 g/L (500 ppm), resulted in 69% increase in biomass productivity and 160% increase in chlorophyll *a* productivity, with respect to control over 10 days [43].

Growth of *Scenedesmus obliquus* was enhanced by 133% at 3.96 g/L (0.5 *v*/*v* %) methanol concentration within 120 h. Assimilation of methanol reached its maximum after 12 h of cultivation and had effect on photosynthetic mechanism, as a 20% decrease in amount of light-harvesting complex II (LHCII) per thylakoid unit was detected after 24 h of cultivation. LHCII is a crucial component of the mechanism responsible for stimulatory effect of methanol, and a lack of this complex caused an inability in MeOH assimilation by *Scenedesmus* mutant cells [44]. Methanol also improved by 100% growth of *Botryococcus braunii* within 10 days [45].

However, inhibitory effect of methanol on microalgae growth has been also numerously reported. Methanol at 3.96 g/L (0.5 *v*/*v* %) caused inhibition of *Chlorella vulgaris* and *Selenastrum capricornutum*, and inhibitory effect was higher for *Chlorella* than for *Selenastrum* [46]. Moreover, methanol caused 50% inhibition of *Raphidocelis subcapitata* [47] and *Chlorella pyrenoidosa* [48], respectively at 4.68 g/L [47] and 6.33 g/L (0.8 *v*/*v* %) [48]. Furthermore, methanol caused 50% inhibition of *Dunaliella tertiolecta*, *Isochrysis galbana* and *Heterosigma akashiwo*, respectively at 23 g/L (23,000 ppm), 21 g/L (21,000 ppm) and 0.5 g/L (500 ppm) [49].

#### 2.2.2. Ethanol

Ethanol undergoes oxidation to acetaldehyde by alcohol dehydrogenase and to acetate by aldehyde dehydrogenase, reactions which take place in mitochondria and/or cytosol [50,51]. Subsequently acetate is converted to acetyl coenzyme A by acetyl-CoA synthetase, and enters tricarboxylic acid (TCA) cycle [50] or glyoxylate cycle [51]. Acetyl-CoA is used for NADH production in TCA cycle [50] or is converted to succinate and malate, by isocitrate lyase and malate synthase in glyoxylate cycle [51].

Ethanol can serve as a carbon source for cultivation of microalgae strains, including photosynthetic protists like *Euglena*. *Euglena gracilis* is a rich source of α-tocopherol, which is synthetized and accumulated in mitochondria and chloroplast, and serve as an antioxidant [52]. Mixotrophic microalgae cultivation, with ethanol as a carbon source and in presence of light, was reported. Ethanol had positive effect on growth of mixotrophically cultivated *Euglena gracilis*, with a 3-fold higher cell number, than in photoautotrophic control. Ethanol also strongly influenced metabolite accumulation in *Euglena* cells, with a 2-fold enhancement in β-carotene and total chlorophyll content, a 2-fold decrease in chlorophyll *a*/*b* ratio and a 7-fold decrease in α-tocopherol, compared to control [53]. Ethanol in presence of light was reported to considerably improve growth and α-tocopherol accumulation in cells of two *Euglena gracilis* strains (a wild strain and a chloroplast-deficient one), with growth improvement and α-tocopherol accumulation higher for the chloroplast-deficient strain, but total biomass and metabolite production higher for the wild strain [52]. *Euglena gracilis* cells cultivated in the presence of ethanol (10 g/L) produced twice more vitamin A and vitamin E than *Euglena* cells cultivated on glucose (10 g/L). However, cell number showed an opposite trend, with the amount of *Euglena* cells twice higher during glucose-based growth, than in ethanol-supplemented culture [54]. Mixotrophic cultivation of *Arthrospira platensis* and *Scenedesmus obliquus* with ethanol as a carbon source resulted in higher biomass production than in photoautotrophic control. Moreover, daily ethanol supplementation further improved *Arthrospira* and *Scenedesmus* biomass production [55,56]. In other studies, cultivation of *Scenedesmus* sp. with 1.42 g/L (0.18 *v*/*v* %) ethanol increased from 50% up to several times biomass concentration, if compared to control [57,58]. The presence of 1.42 g/L (0.18 *v*/*v* %) ethanol also increased lipid and fatty acid content, with a change in fatty acid profile: decrease of saturated fatty acids and increase in polyunsaturated fatty acids [58]. An alteration of fatty acid profile, due to ethanol presence, was also reported for other microalgae. Mixotrophically grown *Nannochloropsis* culture contained higher amount of palmitic acid (C16:0) and smaller amount of oleic acid (C18:1), than photoautotrophic control without ethanol. Additionally, a 1.3-fold stimulation in biomass production and 4-fold increase in respiratory rate was observed [59]. Mixotrophically grown *Chlorella kessleri* culture possesed higher amount of C16:0 and smaller amounts of C16:1 and C16:2, if compared to photoautotrophic control without ethanol. Additionally, a 2.5-fold increase in biomass production was observed [60]. Ethanol also improved growth of *Scenedesmus obliquus*, *Chlorella ellipsoidea*, *Nannochloris* sp., *Gleocystis ampla*, *Navicula saprophila*, *Nitzschia* sp., *Nitzschia dissipata* and *Thalassiosira weissflogii* [61,62].

Microalgae are also capable of using ethanol during heterotrophic cultivation. Ethanol stimulated *Chlorella* growth both in light presence and in dark, with enhancing effect of ethanol being pronounced in the dark [63]. *Euglena gracilis* is also a source of paramylon (β-1,3 glucan), which is synthetized in pyrenoids and accumulated in a form of grains in cytoplasm, as a reserve polysaccharide [64]. Ethanol was successfully used as a carbon source for heterotrophic cultivation of *Euglena gracilis* (a bleached strain) to produce proteins, paramylon or α-tocopherol, and those productions can be influenced by other organic (glutamic acid, malic acid) or inorganic (NH_4_^+^) compounds in a medium [65]. Ethanol improved heterotrophic *Scenedesmus* sp. growth and lipid productivity if compared to photoautotrophic control, although lipid content (% dry weight) decreased [58]. Ethanol was reported to support *Nannochloropsis* sp. growth in the dark, although biomass production was smaller by 32% than during photoautotrophic growth. Moreover, a drop to zero in chlorophyll content, a 3.4-fold increase in respiratory rate, and a change in fatty acid composition: increase in saturated fatty acids (C16:0, C18:0) and decrease in unsaturated fatty acids (C18:1, C20:5), was detected [59]. It was also confirmed that *Crypthecodinium cohnii*, a strict heterotrophic microalga, was able to grow on ethanol to produce docosahexaenoic acid (DHA) [66]. Finally, colorless heterotrophic unicellular alga *Polytomella* spp. was able to grow on 1.84 g/L (40 mM) ethanol as a sole carbon source [67].

Ethanol concentrations within a range of 4-10 g/L can support and stimulate growth of *Euglena* strains (Table S1). Growth of other strains can be supported and/or stimulated at lower ethanol concentrations, up to 3 g/L depending on the strain and the increase in ethanol concentration causes inhibitory effect for microalgal growth. Ethanol at concentrations equal to or higher than 16 g/L, exerted negative effect on *Spirulina platensis* growth, but also on oxygen evolution and cellular respiration [68]. *Synechocystis* sp. growth was also inhibited with the increase in ethanol concentrations. Exposure to ethanol resulted in the alteration in *Synechocystis* cell metabolism, with up-regulation of proteins involved in photosynthesis, oxidative stress response, transporting mechanism or rigidity of cell membranes and envelopes, and down-regulation of proteins related to protein biosynthesis and carbohydrate metabolism [69]. Ethanol at 15.78 g/L (2 *v*/*v* %) caused a 44% inhibition of *Monodus subterraneus* growth [70]. It was also reported that ethanol even at a concentration as low as 0.39 g/L (0.05 *v*/*v* %), could exert inhibition on *Chlorella vulgaris* and *Selenastrum capricornutum* growth, and inhibitory effect was higher for *Chlorella* than for *Selenastrum* [46]. Furthermore, ethanol caused 50% inhibition of *Dunaliella tertiolecta*, *Isochrysis galbana* and *Heterosigma akashiwo*, respectively at 16 g/L (16,000 ppm), 15 g/L (15,000 ppm) and 2.5 g/L (2500 ppm) [49].

#### 2.2.3. Other Organic Solvents

Effect on organic solvents on microalgae depends on solvents concentrations, but solvents type and microalgal strains used are also crucial factors. From ethanol, butanol and hexane tested on *Synechocystis* sp. and *Synechococcus elongatus* growth, hexane showed the highest toxicity, followed by butanol and ethanol. Moreover, *Synechocystis* was more tolerant to ethanol and hexane, and less tolerant to butanol than *Synechococcus* [71]. *Anabaena variabilis* showed much higher tolerance to hexane and heptane than *Monoraphidium braunii*, *Dunaliella salina* and *Chlamydomonas reinhardtii* [72]. Acetonitrile was around 2.5 times more inhibitory for *Raphidocelis subcapitata* (*Pseudokirchneriella subcapitata*) than methanol [47,73]. Acetone at 5.2–6.4 g/L [74,75] and butanone (methyl ethyl ketone) at 8.6 g/L [76], caused 50% inhibition of *Pseudokirchneriella subcapitata* growth. Acetone appeared to be more inhibitory towards *Dunaliella* and *Isochrysis*, if compared to other tested solvents (ethanol, methanol, DMSO, DMF), but methanol was the most toxic to *Heterosigma* [49]. As a contrary, acetone, on a par (*v*/*v* %) with DMSO, was the least toxic solvent towards *Chlorella pyrenoidosa*, followed by DMF, methanol and ethanol [48]. It is consistent with another report, where ethanol was stronger inhibitor of *Raphidocelis subcapitata* growth, than acetone or DMSO [77]. DMF, at different concentrations, was reported to inhibit or stimulate growth of various microalgae species (*H. akashiwo*, *I. galbana*, *D. tertiolecta*, *S. capricornutum*, *C. vulgaris*, *P. subcapitata*) [46,49,73,78,79]. *I. galbana* and *H. akashiwo* were more susceptible to DMSO (3-fold, 4-fold) than *D. tertiolecta* [49]. *Anabaena variabilis* was 2-fold more resistant to DMSO and 3.9-fold more resistant to hexane, when compared to *Anabaena inaequalis* [80]. Among alcohols, decanol showed the highest inhibitory effect on *P. subcapitata* growth, followed by octanol, hexanol, pentanol and butanol [75]. Isopropanol (2-propanol) was reported to effectively suppress oxygen production in *Pseudokirchneriella subcapitata* at a concentration 4 times lower than DMF, and twice lower than methanol (in g/L) [73]. 1-Propanol was nearly twice more toxic towards *Pseudokirchneriella subcapitata* than 2-propanol [74]. On the other hand, *n*-BuOH and *iso*-BuOH caused the same inhibition of *P. subcapitata* growth [75]. Acetaldehyde showed extremely high toxicity to *P. subcapitata* growth, at inhibitory concentration being five orders of magnitude smaller, if compared to acetone or acetonitrile [74].

A case of microalgal strain thriving at high solvent concentation was reported in literature. *Chlorella vulgaris* was able to tolerate isopropanol (IPA) at concentrations up to 16 g/L, with bioconversion of isopropanol to acetone, although nearly 50% inhibition was observed at 16 g/L of IPA [81].

Contrary to species mentioned above, *Polytomella caeca*, a non-photosynthetic unicellular alga, was able to utilize alcohols such as butanol, amyl alcohol and hexanol as sole carbon sources (besides ethanol), at a pH range from 4 to 7 [82].

#### 2.2.4. Glycol Solvents

Glycols are a group of diol solvents that include ethylene glycols (EG), propylene glycols (PG), but also alkyl ethers, such as ethylene glycol monobutyl ether (EGBE). EG and PG were found to exert 50% inhibition of *Selenastrum capricornutum*, respectively at 10.9 and 20.6 g/L [83]. In another study, ethylene glycol caused 50% inhibition of *Pseudokirchneriella subcapitata* at 36.6 g/L [75]. EGBE, also known as 2-butoxyethanol, exerted 50% inhibition on *Pseudokirchneriella subcapitata* at 1.84 g/L [75,84]. Interestingly, propylene glycol at 10 g/L stimulated *Selenastrum* growth, when compared to control [83]. Moreover, EG and PG at lower loadings, 2.59 and 2.1 g/L respectively, were reported to serve as a carbon source for *Chlorella protothecoides* growth [85].

#### 2.2.5. Cyclic Solvents

Cyclic solvents, such as tetrahydrofuran, dioxane, cyclohexane, cyclohexanol and cyclohexanone can exert negative effect on microalgae. Tetrahydrofuran (furanidine) at 2.57 g/L (0.29 *v*/*v* %) caused 50% inhibition of *Chlorella pyrenoidosa* [48]. Dioxane (1,4-dioxane) was reported to cause inhibition of *Scenedesmus quadricauda* and *Microcystis aeruginosa*, respectively at 5.6 and 0.575 g/L [86]. Cultivation of *Chlorella* strain with cyclohexane 1.55 g/L (0.2 *v*/*v* %) resulted in a complete growth inhibition within the first 10 days, but then growth recovery occurred resulting in 130–170% enhancement in microalgal growth at the 25th day of cultivation, when compared to control [63]. In another study, cyclohexane at 19 mg/L caused 50% inhibition of *Pseudokirchneriella subcapitata* growth [75]. Cyclohexanol and cyclohexanone caused 50% inhibition of *Pseudokirchneriella subcapitata* growth, respectively at 0.41 and 1.16 g/L [75]. Interestingly, partial biotransformation (reduction) of cyclohexanone into cyclohexanol in the culture of *Chlorella minutissima*, *Nannochloris atomus*, *Dunaliella parva*, *Porphyridium purpureum* or *Isochrysis galbana*, was reported [87].

#### 2.2.6. Chlorinated Solvents

Chlorinated solvents are a group of solvent containing chloride in their structures and include dichloromethane, trichloromethane, tetrachloromethane, dichloroethane, trichloroethylene, tetrachloroethylene and tertachloroethane.

Chlorinated solvents were reported to cause growth inhibition of numerous green microalgae and diatoms strains [61,62]. Microalgal cell metabolism can be affected due to exposure to chlorinated solvents. Dichloromethane and dichloroethane exerted inhibitory effect on *Chlorella vulgaris* culture, and inhibition was accompanied with the damage of thylacoid membranes, increased amount of starch granules, the alteration of cell shape and the change in transcription of photosynthesis-related genes [88]. On the other hand, dichloromethane and trichloroethylene, at concentrations of respectively 2 µg/L-2 mg/L and 3 µg/L-3 mg/L, did not have any effect on *Chlorella vulgaris* and *Selenastrum capricornutum* growth, but caused death of *Volvulina steinii* culture [89].

According to literature, tetra-chlorinated hydrocarbons are more toxic than tri-chlorinated ones. Tetrachloroethylene appeared to be 10-fold more toxic to *Chlamydomonas reinhardtii*, when compared to trichloroethylene, and tetrachloromethane was found to be around 54 times more inhibitory towards *Chlamydomonas* than trichloromethane [90]. Growth of cyanobacterium *Synechococcus elongatus* was inhibited in the presence of trichloroethylene, tetrachloroethylene and tetrachloroethane, with tetrachloroethylene showing stronger inhibition than trichloroethylene. Additionally, oxidative stress was detected, what was demonstrated by increased level of lipid peroxidation and enhanced activities of peroxidase and SOD [91]. In another study, tetrachloromethane was around 22 times more inhibitory to *Pseudokirchneriella subcapitata* than trichloromethane (chloroform) [75].

*Cis*-*trans* isomerism of chlorinated compounds can also influence toxicity, as *trans*-1,2-dichloroethylene was nearly twice more inhibitory for *Pseudokirchneriella subcapitata*, when compared to *cis*-1,2-dichloroethylene [74].

Although generally inhibitory for microalgae growth, some reports show also stimulatory activity of chlorinated solvents at lower concentrations. For example, growth of *Raphidocelis subcapitata* was significantly stimulated in the presence of trichloroethylene (TCE) at low concentration (0.05–0.1 g/L), and was inhibited at higher TCE concentration (>0.1 or >0.2 g/L) [92]. Also, growth of *Gleocystis ampla* was considerably enhanced at lower tested concentration of trichloroethylene, chloroform and tetrachloromethane [61].

Chlorinated aromatic solvents can affect microalgae cultures. Thus, chlorobenzene, 1,2-dichlorobenzene, 1,2,4-trichlorobenzene and 1,3,5-trichlorobenzene caused 50% inhibition of *Pseudokirchneriella subcapitata* growth, at concentrations 7.8, 2.85, 0.64 and 1.68 mg/L respectively [74]. The presence of organic matter can change the toxicity of chlorinated aromatic solvents. Toxic effects of chlorobenzene and 1,2-dichlorobenzene towards *Chlorella pyrenoidosa* were slightly increased in the presence of Suwannee River Natural Organic Matter (SRNOM) [93]. The isomers of trichlorobenzene were reported to alter structure and composition in diatom *Cyclotella meneghiniana* cells. Exposure to 1,2,4-trichlorobenzene affected mitochondria, vacuoles (autophagic, central, fibrous), nucleus, but also lipids, fatty acid composition, polyphosphate fraction in *Cyclotella* cells, and the positive or negative effects were dependent on exposure time (from 10 min to 5 days) [94]. Treatment of *Cyclotella* culture with 1,3,5-trichlorobenzene resulted in the alteration of chlorophyll *a* content, but also chlorophyll *a*/neutral lipids and neutral/polar lipids ratios, and the effect was dependent on exposure time, the time of adding tested chemical into the culture and temperature of cultivation [95].

Volatility of chlorinated solvents is an important factor affecting microalgal toxicity tests. Removal of volatile solvents causes the decrease in solvent concentration during cultivation time and underestimation of results depicting inhibitory effect of solvents on microalgae growth. Possible solutions to overcome this problem are reduction of cultivation time, analytical control of solvent concentration in a real-time and/or application of closed test systems [16,90]. A type of cultivation systems: open (polystyrene plates) or sealed (glass enclosures) influenced results of microalgal toxicity tests [92]. However, closed test systems cause hindrance in proper gass exchange [90].

#### 2.2.7. Aromatic Solvents

Aromatic solvents contain a benzene ring in their structure, with side groups (methyl, hydroxyl, nitro, nitrile and/or chloride) or are structurally related to benzene, as the case of pyridine.

Benzene, toluene or xylene within a concentration range of 0.1–10 mg/L caused partial inhibition, partial stimulation or no effect towards various microalgae strains such as *Amphidinium carterae* (dinoflagellate), *Skeletonema costatum* (diatom), *Dunaliella tertiolecta* (green microalga) or *Cricosphaera carterae* (coccolithophorid) [96]. In other studies, benzene at 15–124 mg/L and toluene at 14–25 mg/L caused a 50% inhibition of *Pseudokirchneriella subcapitata* growth [74,75]. Xylene, depending on isomeric form (*o*, *m*, *p*), caused 50% inhibition of *P. subcapitata* growth, within 8–26 mg/L [75]. Benzene and toluene at high concentration (5–10%) caused death of green microalgae (*Chlorella*) and diatoms (*Synedra*, *Gomphonema*, *Fragilaria*) in a prolonged cultivation time [97,98]. On the other hand, benzene within a concentration range: 50–100 µg/L, did not cause any relevant change in *Microcystis aeruginosa* growth or intracellular content of microcystin-LR, a peptide toxin produced by *Microcystis* [99].

Ethylbenzene was toxic to *Skeletonema costatum* and *Selenastrum capricornutum*, respectively at 7.5 and 7.2 mg/L after 48 h, at 4.9 and 5.4 mg/L after 72 h or at 7.7 and 3.6 mg/L after 96 h [100]. In another study, *Pseudokirchneriella subcapitata* growth was inhibited by 50% at 1.34 mg/L of ethylbenzene [74].

Industrial spills can contain mixtures of different organic compounds. Therefore, solvent mixtures also should be considered, in terms of their effect on microalgae. A mixture of benzene (52%), toluene (28%), ethylbenzene (5%), *o*-xylene (5%), *m*-xylene (5%) and *p*-xylene (5%), named BTEX, was reported to effectively (50%) inhibit growth of *Selenastrum capricornutum* at 22.7 mg/L [101].

The effect of nitrobenzene (NB) was studied in *Microcystis aeruginosa* cultures. It was observed that NB at 0.2 mg/L was able to inhibit *M. aeruginosa* growth, but also to increase protein productivity and to decrease microcystin-LR productivity in *Microcystis* cells. Additionally, it was concluded that nitrobenzene could undergo biodegradation by *Microcystis aeruginosa* [102,103]. In another study, nitrobenzene at 13.9 mg/l caused a 50% inhibition of *Pseudokirchneriella subcapitata* growth [74].

Benzonitrile caused 50% inhibition of *Pseudokirchneriella subcapitata* growth within concentrations 23–142 mg/L [74,104].

Pyridine, 2-methylpyridine (α-picoline) and 3-methylpyridine (β-picoline) were tested in terms of their inhibitory effect on *Chlorella vulgaris* growth [105]. All three tested compounds showed inhibitory effect on *Chlorella* biomass, protein and chlorophyll content, within 14 days. Interestingly, the presence of α-picoline at a smaller concentration of 0.117 g/L (0.0125%) resulted in *Chlorella* biomass content higher by 67%, if compared to control [105].

Some microalgae are able to open aromatic rings in phenolic compounds via enzymatic cleavage (*ortho* or *meta*) of dihydroxybenzoic derivatives [106].

Cresols, methylated homologues of phenol, are another group of aromatic chemicals, which were tested in microalgae cultures. *Para*-cresol (*p*-cresol), at concentrations: 0.054–0.43 g/L (0.5–4 mM), was used as a carbon source during heterotrophic cultivation of golden-brown microalga *Ochromonas danica*, although the increase in *p*-cresol concentrations resulted in longer lag phase and delayed removal of *p*-cresol from medium [107]. *P*-cresol was also degraded in autotrophic *Scenedesmus obliquus* cultures, with a cleavage of *p*-cresol into phenol and methyl group, the latter one converted to methanol. Methanol and phenol served as carbon sources in autotrophic *Scenedesmu*s cultures, and assimilation of methanol provided energy for phenol fixation. Small *p*-cresol concentration, 0.0162 g/L (0.15 mM), stimulated by 20% *Scenedesmus* growth after a few days of cultivation, with 100% removal of *p*-cresol from medium [108]. In order to be removed from medium, *p*-cresol has to undergone conversion to phenol. It is consistent with observations for *Ochromonas danica* cultures, where time necessary for *p*-cresol removal from cultivation medium was twice longer than for phenol [107]. The presence of carbon (inorganic, organic) can influence the effect of cresols on microalgae growth. *Meta*-cresol or *para*-cresol, 0.162 g/L (1.5 mM), in the presence of glucose, had stimulatory (81% and 48%) effect on *Scenedesmus obliquus* growth, but did not cause any effect when 10% CO_2_ was applied. *m*-cresol and *p*-cresol seemed to stimulate *Scenedesmus* growth, when CO_2_ was not applied or applied at limited mode. Removal of cresols was the highest under CO_2_ limited conditions, and *p*-cresol was biodegraded with a 2-fold higher efficiency than *m*-cresol [109].

### 2.3. Effect of ILs on Microalgae Growth and Cell Metabolism

IL molecules consist of the cationic and the anionic part [47,110,111,112,113,114,115,116,117,118,119,120,121,122,123,124,125,126,127,128,129,130,131,132,133]. ILs containing cations in a form of imidazolium (IM), pyrrolidinium (Pyr[r]) or pyridinium (Py) rings, that possess alkyl and methyl side groups (1-alkyl-3-methylimidazolium, [C_n_MIM]^+^; 1-alkyl-1-methylpyrrolidinium, [C_n_MPyrr]^+^; 1-alkyl-3-methylpyridinium, [C_n_MPy]^+^). Alkyl side groups consist of various (2, 3, 4, 6, 8, 10, 12, 16 or 18) carbon atom numbers, forming ethyl, propyl, butyl, hexyl, octyl, decyl, dodecyl, hexadecyl or octadecyl groups. Some variations in side chains, such as methoxyethyl, methoxyethoxymethyl, diethoxy, hydroxyethyl, chloroethyl, trimethylsilylmethyl, ethoxyphenyl, (ethoxycarbonyl)phenyl, methylendioxyphenylacetate, methylendioxyphenyl(acetoxy)acetate are also reported. Positively charged head groups (ammonium, phosphonium, cholinium) can be also found in ILs. Ammonium and phosphonium groups usually contain four moieties. Cholinium was present in a form of trimethylethanolammonium cation or benzyldimethyl(2-hydroxyethyl)ammonium cation. Mandelic acid IL derivatives can also constitute cationic parts. The most common anions in ILs tested are chloride (Cl^−^), bromide (Br^−^), tetrafluoroborate (BF_4_^−^), bis(trifluoromethylsulfonyl)-imide (Tf_2_N^−^), iodide (I^−^) and hexafluorophosphate (PF_6_^−^). Other ions are hexafluoroantimonate (SbF_6_^−^), lactate (L^−^), tartrate (T^−^) or bicarbonate (Bic^−^), bitartrate (Bit^−^) and dihydrogencitrate (DHCit^−^). All ILs mentioned above were tested in numerous eco-toxicological studies in terms of their effect on microalgae, which are primary producers and play a crucial role in aquatic ecosystems. Both cations and anions in ILs structure can exert effect on microalgae growth (Table S2).

#### 2.3.1. Effect of Cations

A type of structure (imidazolium, pyrrolidinium, pyridinium), in a cationic part, influences the toxicity of ILs towards microalgae. Oxygen evolution in culture media of *Pseudokirchneriella subcapitata* was found to be more inhibited in the presence of C_n_MPyBr than C_n_MIMBr [110]. In another study, C_n_MPyBr was detected to be around 2.5-fold more toxic towards *Pseudokirchneriella subcapitata* than C_n_MPyrrBr [111]. Also pyridinium-based ILs [C_4_Py]Tf_2_N was proved to be more toxic for *Pseudokirchneriella subcapitata* than [C_4_MIM]Tf_2_N, and [C_4_MPyr]Tf_2_N was found to possess much smaller inhibitory activity, if compared to Py and MIM [112]. Alkyl chain of cation group plays an important role in toxicity of ILs. Inhibitory ability of ILs increases when the alkyl chain of the cation part is longer due to increased chain lipophilicity, thereby interacting with phospholipid bilayers or hydrophobic domains of membrane proteins, causing alterations in cell membranes and an increase in membrane permeability [113]. For example, [C_8_MIM]BF_4_ showed a few orders higher toxicity against *Scenedesmus rubescens*, than [C_4_MIM]BF_4_ [114]. In another study, [C_6_MIM]Br was up to 4 times more toxic for *Scenedesmus obliquus* and around 2-fold more toxic for *Chlorella ellipsoidea*, if compared to [C_4_MIM]Br [115]. In another study, toxic effect of [C_8_MPy]Br and [C_8_MPyrr]Br on *Pseudokirchneriella subcapitata* growth was around 200 times higher than [C_4_MPy]Br and [C_4_MPyrr]Br [111]. Toxicity of ILs increases with the increase in the C number of alkyl chain, until the “cut-off” effect appears, where a further increase in C_n_ number fails to enhance toxicity or even toxic effect is alleviated [116]. Except for IM, Py and Pyr structure, also ILs based on ammonium and phosphonium head groups were reported to possess significant toxicity. Tetrabutylammonium tetrafluoroborate, [N_4_,_4_,_4_,_4_]BF_4_, and trihexyltetradecylphosphonium chloride, [(Hex)_3_(TDec)P]Cl, were around 20 times and 4200 times more toxic for *Raphidocelis subcapitata*, than [C_4_MPyr]BF_4_ [47]. A class of methylimidazolium and pyridinium derivatives of methylenedioxy-mandelic acid, posessed various toxicity against *Chlorella vulgaris* and *Pseudokirchneriella subcapitata*, and the difference in structure (MIM vs. Py, methyl vs. butyl esterification, the presence of acetoxy linker) significantly influenced inhibitory effect [117].

#### 2.3.2. Effect of Anions

The anionic part of ILs can also contribute to their toxic effect. Several reports show that ILs possessing the same cations, but differing in anions can exert different effects on microalgae growth. Chol^+^ with BiT^−^ exerted 8.6-fold higher toxicity against *Raphidocelis subcapitata*, if compared to Chol^+^ with Bic^−^ [118]. In a study presenting the effect of various anions in [C_4_MIM]^+^ IL on *Selenastrum capricornutum* growth, SbF_6_^−^ was the strongest toxicant, followed by PF_6_^−^, Br^−^, Cl^−^ and BF_4_^−^. It was suggested that release of F^−^ halide ions could be one of possible reasons for the toxic effect of some fluoride-containing ions [119]. Imidazolium ILs containing NTf_2_^−^ anion, were more toxic for *Scenedesmus vacuolatus*, than imidazolium ILs (with the same side chains) possessing halides (I^−^, Cl^−^, Br^−^) [120]. A change in anion from Br^−^ to I^−^ in [C_2_PhBIM]^+^ IL caused a less than 2-fold increase in toxicity against *Scenedesmus vacuolatus* [121]. Chol^+^ with Cl^−^ exerted slightly higher toxicity against *Raphidocelis subcapitata*, if compared to Chol^+^ with DHCit^−^ [118]. On the other hand, there was no difference between [C_2_ClMIM]Tf_2_N and [C_2_ClMIM]Cl, in terms of their toxicity towards *Pseudokirchneriella subcapitata* [112]. In another study, [MOEMPyr]NTf_2_ and [MOEMPyr]BF_4_ at the same concentrations (g/L) caused the same inhibition of *Raphidocelis subcapitata* growth [122]. Anions such as lactate (L^−^) or tartrate (T^−^), present in ILs under the enantiomeric forms d-(−) or l-(+), were reported to strongly affect toxicity towards microalgae. For instance, a d-(−) enantiomer of [C_2_MIM]L was found to possess more than twice stronger toxicity towards *Scenedesmus obliquus* than an l-(+) form [123]. On the contrary, enantiomeric l-(+) forms of [HMIM]T, [C_8_MIM]T and [C_10_MIM]T caused stronger inhibition of *Scenedesmus obliquus* growth, if compared to d-(−) tartrate forms [124].

#### 2.3.3. Effect of Cultivation Conditions

The effect of ILs on microalgal cultures depends on IL molecular structure, but also exposure time, temperature and presence of other organic and mineral compounds, can be relevant factors.

The toxicity of ILs towards microalgae was enhanced, alleviated or was not influenced by a change in incubation time, depending on IL type used and its concentration [78,111,114,115,125].

Increase in temperature from 25 to 28 °C caused a slight increase in toxicity of [C_n_MIM]Br towards *Chlorella ellipsoidea*. The enhancement of toxicity with temperature increase was suggested to be associated with increased activities of extracellular or intracellular enzymes [115].

The presence of acetone decreased toxic effect of [C_8_MIM]BF_4_ or [C_4_MIM]BF_4_-[C_8_MIM]BF_4_ mixture towards *Scenedesmus rubescens* [114]. Nutrient composition in growth media can affect microalgae response towards ILs. It was reported that *Chlamydomonas reinhardtii*, cultivated in a medium rich in P and N, possessed higher resistance against [(C_4_, C_6_ or C_8_)MIM]Br, if compared to cultivation in groundwater medium, containing major nutrients at low concentrations [126]. Nutrient deficiency could induce a stress in microalgal cells and thus an increased sensitivity towards ILs. Therefore, optimal nutrient levels were suggested to prevent stress in microalgal cells and improve their resistance towards ILs [126]. Salinity is an important factor affecting ILs-microalgae interactions. Increase in salinity was reported to decrease inhibitory effect of [C_n_MIM]Cl on *Chlorella*, *Oocystis* and *Cyclotella* growth, due to complexation of inorganic anions and alkylimidazolium cations, resulting in a limited contact between ILs and microalgal cell surface [127]. A change in salinity also influenced the effect of [C_n_MIM]BF_4_ on *Dunaliella tertiolecta* growth, as well as carotenoid and chlorophyll *a* content in cells [113]. On the other hand, a decrease in salinity did not alter the effect of [C_4_MIM]Cl on *Skeletonema marinoi* [128].

#### 2.3.4. Effect of ILs on Microalgal Cell Wall Structure, Morphology and Metabolism

ILs can exert different effects on microalgae cells according to the structure of cell walls. For instance, structural differences within frustules between *Skeletonema marinoi* and *Phaeodactylum tricornutum* were suggested to be the reason for different sentitivity of these diatom strains to [BMIM]Cl, [MOEMIM]Cl and [M(OE)_2_MIM]Cl [128]. The different structures of cell walls found in microalgal cells, are a key factor in ILs-microalgal cells interactions. *Chlorella vulgaris* cells, have a wall made of cellulose, and they were more resistant against [C_n_MIM]Cl, when compared to *Cyclotella meneghiniana* cells, possessing frustules made of silica [127]. In another study, *Scenedesmus quadricauda* (cellulose cell wall) was more susceptible to [C_n_MIM]Br, than *Chlamydomonas reinhardtii* (glycoprotein cell wall) [126]. On the contrary, cyanobacteria *Geitlerinema amphibium*, with cell wall made of peptidoglycan, showed much lower resistance to [C_n_MIM]Cl, than did green microalgae and diatoms [127]. In another study, toxicity of [C_n_MIM]Cl towards cell wall-possessing *Chlamydomonas reinhardtii* (wild-type) was lower than towards cell wall-lacking *Chlamydomonas reinhardtii* (mutant), suggesting that cell wall serves as a barrier reducing ILs-cell membrane interactions [129]. On the contrary, *Scenedesmus obliquus* (cellulose cell wall) was more sensitive towards enantiomers of [EMIM]L than *Euglena gracilis* (lack of cell wall), as ILs can dissolve cellulose and then damage the integrity of cell membranes [123].

Contact between ILs and microalgae leads to separation of cell wall from cell membranes [125]. Dissolution of cell wall affects membrane integrity and increases permeability, enabling ILs to enter inside the cell and affect intracellular components and metabolism. Exposure to ILs can cause oxidative stress and generation of Reactive Oxygen Species (ROS) that result in lipid peroxidation and production of malondialdehyde (MDA) [130,131,132]. Photosynthetic apparatus in microalgal cells can be negatively affected due to exposure to ILs. Structure of chloroplasts can be damaged, chlorophyll metabolism can be inhibited, chlorophyll content in cells can decrease [125,131,132,133], and Chl *a*/*b* ratio can be altered [124]. Growth inhibition and inhibition of esterase activity in microalgal cells were mentioned during toxic effect of ILs towards microalgae [133]. Also, respiration and energy conversion can be affected by ILs, as swelling of mitochondria was reported in *Scenedesmus obliquus* [125].

Indeed, microalgae possess mechanisms to protect their cells against negative effect of ILs. For instance, oxidative stress in *Synechococcus* sp., *Phaeodactylum tricornutum* and *Skeletonema costatum* cells can be alleviated due to the increase in protein content and activity of antioxidant enzymes such as superoxide dismutase (SOD), catalase (CAT) and peroxidase (POD) [130,131,132]. Chlorophyll and carotenoid contents were also reported to increase in *Dunaliella tertiolecta* cells exposed to ILs. Those pigments possess antioxidant properties, and they can be thus synthetized in abundance to cope with oxidative stress [113]. *Euglena gracilis*, a strain capable of alterating cell shape, was observed to release chloroplasts and shift its growth towards heterotrophism, upon exposure to ILs [123]. Appearance of deposits in vacuoles as a detoxification method in *Scenedesmus obliquus* was also mentioned [125].

Although generally toxic for microalgae, some ILs, such as [BMPyr]Br, [BMIM]Cl, [OMIM]Br can also possess stimulatory effects in microalgae exposed to low concentrations of these ILs, 0.66 g/L (3 mM), 0.174 g/L (1 mM) and 5 mg/L respectively and/or at initial phase of cultivation. As a result of exposure, a slight growth enhancement, a stimulation of esterase activity, an increase in chlorophyll fluorescence and Chl *a* content in microalgae cells, were reported [111,132,133].

## 3. Effect of Organic Solvents on Extraction of Valuable Compounds from Microalgae

Organic solvents can influence growth and metabolism of microalgae, but are also used to extract valuable compounds from microalgal biomass, such as lipids and carotenoids. The mechanism of extraction generally consists of the following steps. Solvent penetrates into microalgal biomass to solvate and separate target product from structural components. Subsequently, solvent in a complex with a product is transferred outside cells via diffusion or exocytosis [134,135].

Production of target compounds from microalgae biomass requires selection of proper solvent and development of efficient extraction techniques. Many organic solvents such as methanol, acetone, ethyl acetate, hexane, chloroform and various ionic liquids ([P(CH_2_OH)_4_][Cl], [BMIM][HSO_4_], [EMIM][DBP]) can be used for lipid and carotenoid extraction from microalgae biomass (Table 1 and Table 2). Nowadays, the attention is focused on increasing the yield of extraction, while reducing extraction steps, energy requirements and process costs. This can be achieved by two diverse, but industrially promising approaches: milking process or selection of classical methods for extraction of harvested biomass, upon cost and production survey.

### 3.1. Milking: Microalgae Extraction During Microalgae Growth

During the traditional extraction processes, cell biomass upon cultivation is harvested and further washed with an organic solvent to destroy cells and release target products out of dead cells. As a consequence, a new microalgal culture is required to produce the next batch of target molecules. To counter the destructive extraction from microalgal biomass, a novel approach has been developed, known as “milking” [136].

Milking is a harvest method of target products from cells which remain viable. This process exists in two methods: spontaneous product secretion from cells into surrounding environment or non-lethal removal of products from cells contacted with biocompatible solvents. During the milking process, cell biomass after solvent treatment is still viable and a new batch of target product can be continuously produced and extracted from solvent-treated cells. The method of simultaneous microalgae cultivation and in situ product extraction from cultivated cells is accomplished in biphasic systems, where an organic solvent is in contact with the cells and it extracts products. After this solvent treatment, the microalgal cells can be further used to produce desirable molecules [137].

In order to repeatedly extract target molecules from continuous viable microalgae culture, the milking process requires the use of a solvent that exerts the lowest possible toxicity on cultivated cells. The most suitable solvents for milking process were reported to be decane, dodecane, tetradecane and hexadecane. These compounds are saturated aliphatic hydrocarbons with log *P* values ≥ 5 and low toxicity towards microalgae, as in opposition to polar solvent such as acetone, diethyl ether, chloroform or dichloromethane, possessing log *P* values < 3 and high toxicity towards microalgae. Decane, dodecane and hexadecane did not cause any decrease in photosynthetic activity in *Dunaliella salina* cells after a 15 min-incubation with the tested solvent. Moreover, the photosynthetic activity in *Dunaliella* cells was even slightly (~10%) increased, upon contact with the solvent, because solvents increased cell membrane permeability, thereby improving the crossing of substrates and products in and out of cells [138]. In another study, dodecane, tetradecane and hexadecane did not exert any negative effect on *Nannochloropsis* sp. cell growth, viability, membrane integrity or dehydrogenase activity, although cultivation time (24–96 h) affected (increased or decreased) activity of dehydrogenase in cells exposed to dodecane [139]. Decane had only a slightly (~5%) inhibitory effect on *Chlorella vulgaris* cell viability after 50 min mixing with the solvent, did not cause ions leakage from cells and even stimulated *Chlorella* growth at shorter (5 min) exposure time [140]. In another study, a type of biocompatible solvent (dodecane, tetradecane) and its concentration (10–20%) had influence on *Chlorella vulgaris* growth. Dodecane at higher concentration (15–20%) stimulated *Chlorella* growth, if compared to control or lower (10%) solvent concentration. On the other hand, tetradecane negatively affected *Chlorella* growth and inhibition increased with the increase in solvent concentration (from 10% to 20%). Similarily to growth, dehydrogenase activity increased with the increase in dodecane concentrations and decreased with higher tetradecane concentrations [141].

Biocompatible solvents possess low toxicity toward microalgae, what renders them suitable solvent to be added into microalgae cultures. However, extraction ability of biocompatible solvents is lower, if compared to polar solvents, what results in the decreased extraction efficiency. The addition of a co-solvent could overcome this problem. A mixture of CH_2_Cl_2_ and decane improved (6 times) the extraction ability, when compared to decane alone [138]. However, toxicity of co-solvent has to be taken into consideration, as addition of CH_2_Cl_2_ to dodecane during *Dunaliella salina* culture, increased cell death [142].

Milking of microalgae was successfully applied and described in a few literature reports. For instance, milking of lipids from *Chlorella vulgaris* was conducted in 4 cycles (7 days each), where a dodecane layer containing the extracted lipids was replaced and the cultivation medium was replenished at every new cycle. The lipid recovery in this process ranged from ~45% (Cycle 1) to ~25% (Cycle 3 or 4) [141]. In another study, a 96-h milking process of lipids from *Nannochloropsis* sp. in the presence of 10% hexadecane (to establish a biphasic system), was achieved. Interestingly, the presence of hexadecane additionally stimulated growth and lipid synthesis in *Nannochloropsis* cells, resulting in 29% higher lipid production, when compared to control without biocompatible solvent [139]. Long-term milking of hydrocarbons from *Botryococcus braunii* during a 6-week continuous cultivation in a bioreactor, was reported. During *Botryococcus* cultivation, the cells excreted hydrocarbons, which were subsequently extracted by hexane, with a very short (15 s twice/day) contact time between solvent and culture. However, only one *Botryococcus* strain tested was resistant to hexane treatment, even under a very short (12 s) contact time [143]. Hexane was reported to be toxic to *Botryococcus braunii*, but could be replaced by heptane, enabling a longer contact time (20 min) without damaging cells [144].

Milking process is a promising extraction method that can improve production of valuable compounds from microalgal cultures. However, this method is still rather in infancy and it may be improved by taking into consideration different parameters: the strain tolerance to particular biocompatible solvent, a localization of target product in cells (inside or outside cells), cultivation parameters (solvent-cell contact time, culture recycling steps), the efficiency of (biocompatible) solvent extraction, the amount of solvent required, the solvent/culture separation and the recovery of the solvent phase.

### 3.2. Extraction Methods and Techniques

Microalgal biomass can be extracted by means of various devices, such as simple tubes [145], Soxhlet apparatus [146,147] or complex reactors [148], where biomass is treated with solvents. Traditional solvents (hexane, acetone, etc.) are commonly used for extraction of lipids and pigments from microalgae, but ILs have potential to serve as a replacement to increase extraction yield [147,149].

As the structure of microalgal cells, especially the rigid cell wall, creates a barrier for product release, solvent extraction is usually accompanied by a variety of additional treatment processes. Mechanical treatment of microalgal biomass, conducted in high pressure homogenizer or bead-milling, is the initial and often crucial step before extraction. As this process generates shear forces, it leads to cell wall degradation and cell rupture, and so to the increased accessibility of the solvent to the cell interior, improving extraction efficiency [150,151,152].

It was reported that high-pressure homogenization improved lipid extraction yields from *Scenedesmus* sp., with a reduction in time and decrease in temperature, if compared to control without mechanical treatment [153].

In order to further increase extraction yield of lipids and carotenoids from microalgae biomass, a series of various physico-chemical extraction techniques such as ultrasound, microwave heating, supercritical fluids, etc., can be applied [154,155].

Ultrasound treatment is a technique providing sounds of high frequency (>20 kHz) which are transmitted to liquid and create regions of alternating pressure, resulting in formation of gas bubbles in the process called cavitation [154]. When the generated bubbles come into contact with the surface of plant material and implode, the high pressure and temperature created locally destroy the structure of plant materials. This treatment is the most common technique used on laboratory scale to support extraction with solvents [156]. Ultrasound treatment was applied during lipid extraction from *Chlorella* sp. [157], pigments from *Cylindrotheca closterium* [158] and fatty acids and pigments from *Chlorella vulgaris* [159].

Microwaves are electromagnetic waves with frequency between 300 MHz and 300 GHz, which are generated by electric and magnetic fields. Microwaves induce, dipole rotation and ion migration in solvent and in structural molecules, resulting in heating, in cleavage of chemical linkages, in increased pressure and rupture of plant material from inside and finally in release of the interior product to the surroundings [160,161,162]. Microwave treatment was applied during lipid extraction from *Isochrysis galbana* [162], *Chlorella* sp. [157] and *Chlorella sorokiniana* [163] and pigments from *Cylindrotheca closterium* [158] and *Phaeodactylum tricornutum* [164], with a great improvement in product extraction yield [163].

Supercritical fluid extraction is a process, where solvents are used under their critical state. Supercritical liquids appear when temperature and pressure are above the critical threshold, at which fluids possess gas-like properties. Supercritical liquids have densities similar to liquids, but decreased viscosities and enhanced diffusivities like gasses, thereby possessing higher power to penetrate into biomass and extract products when compared to conventional liquids [165]. For carotenoid production, the commonly used supercritical fluid is CO_2_ [166], together with the addition of organic solvents [167,168], to enhance the extraction.

### 3.3. Energy and Production Cost Study

The extraction technologies described above are based on the use of various solvents, most of the processes being demonstrated only at a lab-scale. The concept of microalgal biorefinery is getting more and more attention and sustainable and economically feasible high-yield processes have become the major focus of microalgal research [169]. To be able to use a proper method at an industrial scale, an efficient, cost effective and environmentally friendly extraction technology has to be developed to extract demanded products. Therefore, in order to select the efficient extraction technology for target products, fundamental energy and production cost studies were performed for representative literature data concerning lipids (Table 1) and various carotenoids (fucoxanthin, β-carotene, astaxanthin) (Table 2) extraction methods. The calculation methods of energy requirement and production cost are provided in S3-Calculation scheme.

#### 3.3.1. Lipid Extraction

The calculated specific energy requirement and the specific production costs for lipid extraction are listed in Table 3, with perceptual structure of energy demand and production costs included. Generally known, microalgal biomass undergoes a pretreatment at the first stage of extraction to open microalgae structure. High pressure homogenizer, microwave or ultrasound can be applied for biomass pretreatment [153,157]. Such a pretreated biomass is consequently mixed with solvent and lipids are extracted. Solvent is finally evaporated, condensed and re-used. Techniques such as microwave treatment can be also used during solvent extraction to enhance lipid yield [163]. For lipid extraction, the lowest energy requirement 137–165 MJ·kg^−1^ and separation cost 0.8–0.92 Eur·kg^−1^ of product was found for the extraction process with pretreatment proposed by [153]. The price of chemicals per 1 kg of product is presented also for illustration (recovery of solvents was excluded/no recovery of solvents was taken into account). The microwaves and ultrasounds were used as pretreatment methods in extraction process presented by [157]. In this case the higher energy demand of both pretreatment techniques is not accompanied by higher yield, thus separation cost is approx. 16-50 times higher in comparison with [153].

#### 3.3.2. Carotenoid Extraction: Fucoxanthin

Fucoxanthin extraction technology was proposed by [158]. For fucoxanthin extraction the lowest energy requirement (127 GJ·kg^−1^) and production costs (704 Eur·kg^−1^) of product were found for the extraction process at ambient temperature without microwave or ultrasound techniques applied (Table 4). The price of chemicals per 1 kg of product is presented also for illustration (recovery of solvents was excluded). Both microwave and ultrasound techniques, used during solvent extraction, did not contribute to the increase of fucoxanthin yield, however these techniques increased the separation cost 1.6–2 times.

#### 3.3.3. Carotenoid Extraction: β-carotene

The specific energy requirement and the specific production cost for supercritical extraction of β-carotene and carotenoids were evaluated (Table 5), with perceptual structure of energy demand and production costs included. The price of chemicals per kg of product is presented also for illustration. For β-carotene extraction process proposed by [167], the lowest energy requirement (65.2 GJ·kg^−1^) of product and separation cost 370 Eur·kg^−1^ occurred at extraction temperature of 40 °C and pressure of 40 MPa with the mixture of CO_2_ and ethanol. When the pure CO_2_ is used as the extraction solvent the lowest energy requirement and separation cost was found for the extraction process occurring at extraction temperature of 60 °C and pressure of 40 MPa. As expected, the solvent compression represents the largest share of the costs for supercritical extraction.

#### 3.3.4. Carotenoid Extraction: Astaxanthin

The specific energy requirement and the specific production cost were evaluated (Table 6) for astaxanthin extraction process proposed by [149]. Perceptual structure of energy demand and production costs are included. The price of chemicals per kg of product is presented also for illustration. Extraction process using combination of ionic liquid in pretreatment step and ethyl acetate in extraction step was found to improve astaxanthin yield in relation to energy demand and production cost, if compared to combination of acetone (pretreatment) and ethyl acetate (extraction). The lowest energy requirement 14 GJ·kg^−1^ of product and separation cost 83 Eur·kg^−1^ was found for the extraction process occurring at temperature of 45 °C pretreated by ionic liquid EMIM DBP and extracted in ethyl acetate.

## 4. Strategies for Organic Solvent Use During Microalgal Cultivation or Extraction

This review evaluates the effect of various organic solvents on microalgae growth and metabolism, as well as extraction of valuable compounds from living and dead microalgae cells. Organic solvents can exert positive or negative effect on microalgae growth, what is crucial when solvent-containing effluents are to be used as a feedstock for microalgae cultivation. Composition of solvents in industrial effluents strictly determines the application of these effluents for microalgae cultures. Methanol and ethanol are organic solvents that at lower loadings can efficiently improve growth of various microalgae strains, as described above (Section 2.2.1 and Section 2.2.2). A stimulatory range (Table 7) for methanol was reported to be 4–8 g/L for *Chlorella* strains, although lower and higher methanol concentrations improved *Chlamydomonas* (1.6 g/L) and *Botryococcus* (23.7 g/L) growth. A stimulatory range for ethanol was reported to be at higher concentrations (4.6–10 g/L) for *Euglena* strains, and at lower ones for *Scenedesmus* strains (0.4–1.8 g/L), *Chlorella* strains (0.4-2.3 g/L) and other strains such as *Arthrospira* (0.15–1.21 g/L) and *Nannochloropsis* (1.38 g/L). Industrial wastewaters and other effluents, containing these solvents within stimulatory ranges, could potentially improve biomass productivity of suitable microalgal strains. Methanol improved the productivity of some proteins [42], lipids [41] and pigments [43] and ethanol contributed to increase the amount of tocopherol [52], of some pigments [53], and lipid content [58,60], showing that these solvents can contribute not only to biomass increase, but also to increased production of target compounds. Ethanol was also reported to affect nucleic acids in microalgae cells. Ethanol increased nucleic acid (DNA, RNA) content in *Dunaliella viridis* cells [170], and ethanol carbon was incorporated into the composition of DNA and RNA in *Chlorella vulgaris* cells [171]. Further, the presence of methanol resulted in the alteration in fatty acid [41] and amino acid [42] profile in microalgal cells. The presence of ethanol increased intensity of protein, phospholipid, nonesterified fatty acid and steroid ester excretion from microalgae cells [170]. The accumulation of triacylglycerides in microalgae cells was also reported, although accompanied with growth inhibition [172]. Therefore, solvents could be possibly used to “design” a desirable profile of target products such as fatty and amino acids, obtained from microalgal cultures.

There are different microalgal cultivation systems, such as closed photobioreactors [6,173] or open systems [6,174,175], the latter ones used commercially [6]. A lack of sterility in open systems can constitute a barrier for using methanol or ethanol, due to the presence of bio-contaminants (bacteria, yeast), which can outcompete microalgae for carbon sources. Nevertheless, methanol was reported to be successfully used as a carbon feedstock to support *Chlorella* biomass production during long-term (45 days) outdoor cultivation, and addition of methanol was regarded as a factor maintaining sterility [41]. Contrary to methanol, which can stimulate microalgae growth only in the presence of light, ethanol was reported to serve as a carbon source also in dark, during heterotrophic cultivation. Therefore, heterotrophic production of microalgal biomass could be carried out in closed stirred tanks sterilised by heat [176] and supplied periodically with filtered ethanol-containing effluent dosages. A possible strategy that could be applied to non-sterile outdoor systems is to maintain such cultivation conditions, which would prevent development of bio-contaminants. Maintaining alkaline conditions (pH = 11) was reported to prevent development of bacteria and a loss of ethanol during outdoor cultivation of ethanol-producing *Synechocystis* sp. [177]. However, it should be remembered, that extreme cultivation conditions can also have inhibitory effect on microalgal cultures.

However, solvents at higher concentrations can exert inhibitory and toxic effect on microalgae. Toxic effect of solvent on microorganism cells, including microalgae, can be expressed in a form of enzyme inactivation, breakdown of transport mechanisms, inhibition of cellular division and cell lysis [178]. A loss of microalgae cell mobility was also observed [170].

Methanol, within a concentration range of 0.5–82 g/L and ethanol at concentrations 1.4–16.5 g/L, caused inhibition of various microalgae strains (Section 2.2.1 and Section 2.2.2).

Acetone, acetonitrile, hexane, DMSO and DMF did not improve microalgae growth, with one exception for DMF [46], and were neutral and/or inhibitory at various concentrations. Hence, the presence of these solvents in industrial wastewaters would not be beneficial for microalgae growth. Higher alcohols (C_n_, *n* ≥ 3), also caused inhibition of microalgae growth, and inhibitory effect increased drastically, with the carbon number in the alcohol molecule [75], although *Polytomella* strain was reported to assimilate alcohols (C_4_, C_5_, C_6_) as carbon sources in dark [82].

Aromatic solvents caused inhibition and death of microalgae cultures with increasing solvent concentrations. Among aromatic compounds, cresol is a solvent that at small concentrations (16–160 mg/L), can support microalgae growth, although stimulatory effect depends on isomeric form of this compound, cultivation conditions (presence of inorganic and organic carbon) and time exposure [107,108,109]. Presumably, industrial effluents containing cresols [179], could be used as a feedstock for microalgae growth.

Chlorinated solvents show a very broad range of inhibitory concentation, from 2 µg/L to 2.86 g/L, depending on the solvent, the microalgae strain and the specificity (open vs closed) of the cultivation system (Section 2.2.6). Inhibitory activity of chlorinated alkane and benzene compounds increases with the increase in the number of chlorine atoms in a molecule [74]. Some chlorinated solvents at small concentrations were also reported to possess stimulatory effect on microalgae growth. Application of chlorinated solvents such as trichloroethylene, chloroform or tetrachloromethane, to stimulate microalgae growth [61,92] could be an interesting approach for increasing the yield of microalgal biomass production. However, only a limited number of strains is capable of tolerating chlorinated solvents and a mechanism of growth stimulation is unknown.

Glycol solvents (EG and PG) are not strong inhibitors of microalgae growth, within the inhibitory range 10–36 g/L, although alkyl glycol ethers showed higher toxicity [75,83,84]. Moreover, at smaller concentrations, EG and/or PG could be potentially used as a carbon feedstock to support growth of some microalgae strains during phototrophic and/or heterotrophic cultivation [83,85].

Because organic solvents at elevated concentrations cause negative effect on microalgae, effluents containing traditional organic solvents at high concentrations can suppress microalgal cultivation. The possible solution to overcome the toxicity of solvents towards microalgae could be the use of strains isolated from the solvent-contaminated environment and possessing higher solvent tolerance [81]. Another possibility could be applications of smaller effluent dosages, to maintain solvent concentrations within stimulatory range during microalgae cultivation.

Industrial effluents containing organic solvents might possess potential to be used as a feedstock for microalgae cultivation. However, only a limited number of solvents showed to support microalgal cultivation (Table 7), and other solvents would be considered as pollutants. Wastewaters can constitute a source of organic carbon and nutrients (N and P) for microalgae cultures [8,11,174], but the presence of organic solvents as pollutants, can have detrimental effect on microalgae. Moreover, industrial effluents and wastewaters contain other components such as sulfur compounds [26], desinfectants and heavy metals [180] that could also affect microalgae cultures.

ILs can exert significant effect on metabolism, morphology and structure of microalgal cells. ILs were reported to cause a damage to cell structure (wall, membranes, organelles), generation of ROS, degradation of lipids, alteration in pigment content, induction of enzymes participating in antioxidant defence system, as well as affecting photosynthesis and respiration mechanisms (Section 2.3.4). Ionic liquids were more toxic for microalgae than traditional, non-chlorinated, non-aromatic solvents, and their inhibitory effect is vastly dependent on IL structure. Among ILs, the most toxic representatives are methylimidazolium salts (chloride) possesing a long (C_10_–C_18_) alkyl side group and being toxic to microalgae within a range 0.07–40 µg/L [116,120,125], as well as some other ILs, such as aryl alkyl imidazolium halides or trihexyltetradecylphosphonium chloride, exerting inhibition on microalgae, respectively at 10–14 [121] and 84 µg/L [47]. The presence of these ILs in wastewater streams used for microalgae cultivation should be avoided and/or monitored, because these ILs can suppress microalgae growth even at very small concentrations.

Solvents, at low concentrations, can improve microalgae growth in some cases, but at high concentrations are used for extraction of valuable components from microalgae cells. Milking is an interesting extraction approach, where solvents remove valuable compounds from microalgal cells without causing lethal effect, so that microalgae culture can be reused for further production of target compounds. This approach requires using proper solvents that can extract molecules from cells without causing cell death. Hydrophilic solvents are too toxic for a milking process, as these solvents were reported to increase membrane permeability, decrease dehydrogenase activity and cause K^+^ leakage in microalgae cells [139]. As a replacement, hydrophobic long chain hydrocarbons showed to be biocompatible solvents, suitable for simultaneous cultivation and extraction. Milking can simplify product extraction from microalgal culture because microalgae cultivation and product extraction can be obtained at the same time without the necessity of harvesting and destroying cells. However, this approach is still in development and a focus should be put on improving biomass productivity and extraction yield. Possibly, combining a traditional solvent as an organic carbon for microalgal biomass growth stimulation and a biocompatible solvent to extract target product from microalgae cells, in a biphasic: aqueous (medium with organic solvent for growth)–biocompatible solvent (for extraction) system, could improve production of target compounds during milking process.

So far, classical extraction approach (Table 1 and Table 2) depending on treatment of microalgal biomass with various solvents and resulting in microalgae death has been commonly used. Such an approach is based on chemical or physical techniques that are applied before or during extraction process to favour cell structure disruption, increased permeability and improved solvent diffusion. As a result, decrease in reaction time, reduction in the use of organic solvents and overall increase in the extraction yield should be achieved. Improvement in production yield in relation to production cost is a mandatory factor, when considering microalgal biomass as a source of potential industrial products. In this review, the specific energy requirement and specific production costs of valuable chemicals isolated by extraction process were calculated (Section 3.3) for representative literature reports. Based on reviewed literature data and performed energy and production cost calculations, it can be stated that the right choice of pretreatment and solvent have a crucial impact on specific energy requirement and specific production costs.

The energy requirements of extraction technology, including substrate’s pretreatment, are crucially influenced by microalgae pretreatment aiming to open microalgae’s structure and increase significantly extraction yield of demanded product. Chemicals are extracted from non-pretreated or preatreated substrates. The following techniques are usually used in laboratory scale for pre-treatment: high pressure homogenizer, microwave or ultrasound pretreatment. Nevertheless, the industrial processing technologies are aiming to use both efficient and energy least demanding techniques. Microwave and ultrasound pretreatments have the highest energy demand, and are thus the most expensive technologies. In contrast, high-pressure homogenizer is among the least energy-demanding pretreatments. Application of high pressure homogenizer as the least energy demanding pretreatment seems to be the best solution for its usage at pilot and industrial scales.

It was found that specific production cost strongly depends on energy demand for solvent recovery and its price. The mixture of chloroform and methanol seems to be the best for lipid extraction in relation to minimum energy and separation cost demand [153]. Whereas in the case of fucoxanthin extraction, the lowest production cost was found for acetone as extractive solvent without any pretreatment [158]. For β-carotene extraction, the use of mixture of CO_2_ and ethanol as the solvent at temperature of 40 °C and pressure of 40 MPa was the most interesting concerning the energy demand and the separation costs [167]. For astaxanthin extraction with ethyl acetate, the process using a IL for pretreatment was found to be less energy-demanding and so to have low production costs, when compared to using pretreatment with acetone [149]. The cost of mixing during extraction was found to be negligible.

Solvent separation represents the main part of separation cost. The same amounts of product can be reached using traditional solvents or ILs. If traditional solvents are used, the energy demand for their evaporation and subsequent recovery remains low. If ILs are used as solvents, their boiling point is typically up to 340 °C at ambient pressure 1030 hPa. Pressurized steam has to be used for their evaporation and corrosion resistant materials have to be used for the evaporator and condenser. This all leads to design of high pressure vessels for solvent separation streams. This makes the extraction technology much more expensive in comparison with traditional solvents that can be separated under ambient temperatures. Efficiency of solvent recovery also plays a very important role, as only pure ones can be reused in extraction technology. Nevertheless, there is a limitation due to their life time, and sometimes a volume of new solvent must be added to the system. The price of traditional solvents is low whereas the purchase price of ILs is very high. The application of traditional solvents seems to be therefore the best solution as for energy and production costs of product, although the use of smaller amount of ILs, combined with higher extraction yield, can make the use of ILs in extraction process more favourable than traditional solvents [147].

The selection of a solvent (highly toxic vs. less toxic) is also important for further utilization of post-extracted microalgal biomass. De-lipided microalgal biomass can be used for biogas (CH_4_) production via anaerobic digestion process. However, remnants of solvent (chloroform, acetone, *n*-hexane) present in microalgal residues, can have inhibitory effect on microorganisms involved in biogas production [181,182,183]. The use of green biodegradable solvents as an alternative to traditional solvents, could be considered [184].

A microalgal-based biorefinery concept requires efficient use of microalgae biomass through optimization of biomass production and extraction process, as well as generation of target products with diverse applications for numerous branches of industry [185]. Optimization of cultivation conditions to favour the accumulation of target products in microalgae cells before extraction process, can be a method to improve the extraction yield [186]. Moreover, cultivation conditions [187] and extraction process parameters [188], including solvent type [159], can also influence comprosition of obtained extracts [159,187,188], that should find applications not only in refinery, but also in food, pharmaceutical and cosmetic industry [185,188].

## Figures and Tables

**Table 1:** Effect of different techniques, process parameters and solvents on lipid extraction yields from microalgal biomass.

Product	Strain	Solvent	Parameters	Yield	Reference
Lipids	*Scenedesmus* sp. (freeze-dried)	Chloroform:Methanol (2:1, *v*/*v*)	Pretreatment: High Pressure Homogenizer, Pressure (1200 psi). Extraction: 1 g sample per 30 mL solvent, 30 min, 35 °C, 500 rpm.	24.9% (*w*/*w*)	[153]
			Pretreatment: none. Extraction: 1 g sample per 30 mL solvent, 5 h, 65 °C, 500 rpm.	19.8% (*w*/*w*)	
			Additional processes: centrifugation, drying.		
Lipids	*Nannochloropsis oculata* (freeze-dried and ground)	Hexane	Soxhlet extraction: 1 g biomass in a thimble, 200 mL solvent in a flask, 80 cycles within 7 h.	9.1% (on dry weight basis)	[147]
		[P(CH_2_OH)_4_]Cl (80% in water)	Extraction: 1 g biomass for 10 mL ionic liquid, 100 °C, 24 h, magnetic stirring. Further, methanol and hexane used to purify lipid fraction.	12.8% (on dry weight basis)	
			Additional processes: centrifugation, rotary evaporation.		
Lipids	*Chlorella* sp. (freeze-dried)	CH_2_Cl_2_/MeOH/Microalgal solution (50 mL/25 mL/20 mL)	Pretreatment: 0.5 g in 20 mL water, stirring for 2 min, ultrasonic waves (40 kHz, 200 W, the actual heating power = 48 W) for 1200 s. Extraction: stirring for 62 min, at room temperature, further 25 mL CH_2_Cl_2_ and 25 mL H_2_O added and a mixture was stirred again.	11.6% (wt %)	[157]
			Pretreatment: 0.5 g in 20 mL water, stirring for 2 min, microwaves (2450 MHz, 530 W, the actual heating power = 380 W) for 75 s. Extraction: stirring for 62 min, at room temperature, further 25 mL CH_2_Cl_2_ and 25 mL H_2_O added and a mixture was stirred again.	11.6% (wt %)	
			Additional processes: centrifugation, rotary evaporation.		
Lipids	*Chlorella sorokiniana*	[BMIM][HSO_4_] 1 g biomass: 5 g solvent	Microwave irradiation 800 W, 120 °C, 60 min.	23% (*w*/*w*)	[163]
			Oil bath: 120 °C, 60 min.	1.1% (*w*/*w*)	
			Additional processes: addition of distilled H_2_O and *n*-hexane, mixing, filtration, evaporation.		

**Table 2:** Effect of different techniques, process parameters and solvents on carotenoid extraction yields from microalgal biomass.

Product	Strain	Solvent	Parameters	Yield	Reference
Fucoxanthin	*Cylindrotheca closterium* (diatom)	Acetone 100% (50 mg freeze-dried biomass per 30 mL acetone)	Room Temperature Extraction (20 °C) 60 min under magnetic stirring	0.45%	[158]
			Microwave Assisted Extraction (56 °C, atm pressure) 5 min, 50 W under magnetic stirring	0.42%	
			Ultrasound Assisted Extraction (8.5 °C) 5 min, 12.2 W under magnetic stirring	0.34%	
			Additional processes: centrifugation, evaporation, purification (chromatography).		
β-carotene	*Synechococcus* sp. (cyanobacterium)	Supercritical CO_2_ (4.6 g homogenized biomass in an extractor with maximal capacity 10 mL) with a flow 0.8 g/min	CO_2_ extraction (3 h)		[167]
			40 °C 200 bar	0.016%	
			40 °C 400 bar	0.035%	
			60 °C 400 bar	0.046%	
			CO_2_ extraction with 5% (vol) ethanol (3 h)		
			40 °C 200 bar	0.036%	
			40 °C 400 bar	0.077%	
			60 °C 400 bar	0.060%	
Astaxanthin	*Haematococcus pluvialis*	Ethyl acetate 2 mL (two rounds) for solvent treated biomass	Biomass (10 mg) treated with [EMIM][DBP] (2.1 mL) at 25 °C for 90 min.	36% of total astaxanthin	[149]
			Biomass (10 mg) treated with [EMIM][DBP] (2.1 mL) at 45 °C for 90 min.	70% of total astaxanthin	
			Biomass treated with acetone at 25–45 °C for 90 min.	~4% of total astaxanthin	
			Additional processes: centrifugation, mixing.		

**Table 3:** Energy requirement, energy and production costs for lipid extraction.

Data Source	[153]		[147]		[157]		[163]	
Strain	*Scenedesmus* sp. (freeze-dried)		*Nannochloropsis oculata* (freeze-dried and ground)		*Chlorella* sp. (freeze-dried)		*Chlorella sorokiniana*	
Pretreatment	no	High pressure homogenizer	no	no	ultrasound	microwave	no	no
Extraction	yes	yes	yes	yes	yes	yes	microwave	solvolysis at high temperature in oil bath
Solvent	Chloroform methanol	Chloroform methanol	hexane	ionic liquid THPC	Dichloremethan methanol	Dichloremethan methanol	ionic liquid BMIMHSO4	ionic liquid BMIMHSO4
w_dB_ (% wt.)	100	100	100	100	100	100	100	100
Y_product_ (% dry wt.)	19.8	24.9	9.1	12.8	11.6	11.6	23.0	1.1
E_SEP_ (MJ·kg^−1^ product)	165	137	987	440	5637	2185	12,700	5550
Pretreatment (%)	0	4.4	0	0	73.4	31.4	98.2	17.0
Mixing (%)	0.5	< 0.1	1.8	1.0	<0.1	< 0.1	<0.1	<0.1
Evaporation (%)	49.8	47.8	49.1	49.5	13.3	34.3	0.9	41.5
Condensation (%)	49.8	47.8	49.1	49.5	13.3	34.3	0.9	41.5
C_SEP_ (Eur·kg^−1^ product)	0.92	0.8	5.6	2.5	49	15	123	35
Pretreatment (%)	0.0	7.4	0.0	0.0	82.9	44.6	99	26.5
Mixing (%)	0.9	<0.1	3	1.8	<0.1	0.1	0.0	0.1
Evaporation (%)	84.8	79.2	83.1	84	14.6	47.3	0.9	62.8
Condensation (%)	14.3	13.4	14	14.2	2.5	8	0.1	10.6
C_CHEMICALS_ (Eur·kg^−1^ product) *	697	555	45,100	21,170	6850	6850	11,000	230,000

* recovery of solvents was excluded.

**Table 4:** Energy requirement, energy and production costs for fucoxanthin extraction.

Data Source	[158]		
Strain	*Cylindrotheca closterium* (diatom)		
Extraction	ambient solvolysis	microwave	ultrasound
Solvent	acetone	acetone	acetone
w_dB_ (% wt.)	100	100	100
Y_product_ (% dry wt.)	0.45	0.42	0.34
E_SEP_ (GJ·kg^−1^ product)	127	207	189
Pretreatment (%)	<0.1	34.5	11.4
Mixing (%)	0.2	<0.1	<0.1
Evaporation (%)	49.9	32.8	44.3
Condensation (%)	49.9	32.8	44.3
C_SEP_ (Eur·kg^−1^ product)	704	1 450	1 140
Pretreatment (%)	<0.1	48.0	18.4
Mixing (%)	0.4	<0.1	<0.1
Evaporation (%)	85.2	44.5	69.8
Condensation (%)	14.4	7.5	11.8
C_CHEMICALS_ (Eur·kg^−1^ product) *	386,700	414,300	511,800

* recovery of solvents was excluded.

**Table 5:** Energy requirement, energy and production costs for β-carotene extraction.

Data Source	[167]					
Strain	*Synechococcus* sp. (cyanobacterium)					
Temperature (°C)	40	40	60	40	40	60
Pressure (MPa)	20	40	40	20	40	40
Pretreatment	no	no	no	no	no	no
Solvent	Carbon dioxide	Carbon dioxide	Carbon dioxide	Carbon dioxide ethanol	Carbon dioxide ethanol	Carbon dioxide ethanol
Solvent flowrate (g/min)	0.8	0.8	0.8	0.8	0.8	0.8
x_ethanol_ (% mol)	0	0	0	5	5	5
w_dB_ (% wt.)	100	100	100	100	100	100
Y_product_ (% dry wt.)	0.016	0.035	0.046	0.036	0.077	0.060
E_SEP_ (GJ·kg^−1^ product)	268.0	143.8	108.3	119.3	65.2	82.8
Solvent compression (%)	50.7	50.7	51.2	50.8	50.8	51.3
Solvent cooling (%)	49.3	49.3	48.8	49.2	49.2	48.7
C_SEP_ (Eur·kg^−1^ product)	1532	816	619	682	370	474
Solvent compression (%)	86.3	86.2	86.5	86.3	86.3	86.6
Solvent cooling (%)	13.7	13.8	13.5	13.7	13.7	13.4
C_CHEMICALS_ (Eur·kg^−1^ product) *	352,170	160,990	122,490	277,760	129,860	166,650

* recovery of solvents was excluded.

**Table 6:** Energy requirement, energy and production costs for astaxanthin extraction.

Data Source	[149]		
Strain	*Haematococcus pluvialis*		
Pretreatment solvent	EMIM DBP	EMIM DBP	Acetone
Temperature (°C)	25	45	25-45
Time (min)	90	90	90
Extraction solvent	Ethyl acetate	Ethyl acetate	Ethyl acetate
w_dB_ (% wt.)	100	100	100
Total astaxanthin (% wt.)	3.2		
Y_product_ (% of total wt. of astaxanthin)	36	70	4
E_SEP_ (GJ·kg^−1^ product)	26	14	277
Pretreatment (%)	1.7	7.5	1
Mixing (%)	0.1	0.1	0.1
Evaporation (%)	49.1	46.2	49.4
Condensation (%)	49.1	46.2	49.4
C_SEP_ (Eur·kg^−1^ product)	144	83	1542
Pretreatment (%)	2.9	12.5	1.8
Mixing (%)	0.2	0.2	0.2
Evaporation (%)	82.9	74.7	83.9
Condensation (%)	14	12.6	14.1
C_CHEMICALS_ (Eur·g^−1^ product) *	4078	2093	15,025

* recovery of solvents was excluded.

**Table 7:** Application range of some solvent types for microalgae cultivation.

Solvent Type	Stimulatory Range	Inhibitory Range
Methanol	1.3–7.92 g/L, ~23 g/L [45]	0.5–82 g/L
Ethanol	0.15–10 g/L	1.4–16.5 g/L
Cresols	16–160 mg/L	n.d.
Chlorinated solvents	0.05–0.1 g/L	2 µg/L–2.86 g/L
Glycols (EG, PG)	~2.5 g/L	10–36 g/L

## Supplementary Materials

Supplementary materials can be found at www.mdpi.com/1422-0067/18/7/1429/s1.
