# Supplementary material for: Effect of Organic Solvents on Microalgae Growth, Metabolism and Industrial Bioproduct Extraction: A Review
Source: Int J Mol Sci. 2017 Jul 4;18(7):1429. doi: 10.3390/ijms18071429 (PMC5535920; doi:10.3390/ijms18071429)
Supplement: Supplementary file 1 [file ijms-18-01429-s001.pdf]

Effect of Organic Solvents on Microalgae Growth, Metabolism and Industrial Bioproduct Extraction: A Review

Krystian Miazek, Lukas Kratky, Radek Sulc, Tomas Jirout, Mario Aguedo, Aurore Richel and Dorothee Goffin

Table S1. Effect of organic solvents and cultivation parameters on microalgae growth and metabolism.

Strain	Solvent	Concentration	Exposure time	Effect on growth	Effect on metabolism	Ref.
		Polar/non-polar solvents (non-chlorinated, non-aromatic)				
Selenastrum capricornutum	DMF	1.27-2.31 g/L (17.4-31.6 mM)	96h	50% inhibition	n.d.	[78]
Selenastrum capricornutum	DMF	0.094-0.94 g/L (0.01-0.1 v/v %)	14 days	Slight stimulation observed	n.d.	[79]
Pseudokirchneriella subcapitata	Methanol<br>DMF<br>Isopropanol<br>Acetonitrile	82 g/L (2570 mM)<br>152.5 g/L (2089 mM)<br>35.4 g/L (589 mM)<br>34 g/L 832 mM	up to 2 h	50% inhibition[PA]	Decreased oxygen evolution rate	[73]
Raphidocelis subcapitata	Acetonitrile<br>Methanol	1786 mg/L<br>4686 mg/L	72h	50% inhibition	n.d.	[47]
Botryococcus braunii	Methanol	~23 g/L (3%)	10 days	100% stimulation	n.d.	[45]
Chlamydomonas reinhardtii	Methanol	1.6 g/L (50 mM)	6 days	35% stimulation	Protein content-(30% increase)[20h] Free amino acid content-(31% increase)[5h] A change in amino acid composition[5h]	[42]
Chlorella minutissima	Methanol	3.96 g/L (0.5 v/v %)	6 days<br>9 days<br>11 days	45% stimulation[A]<br>27% inhibition[A]<br>74% inhibition[A]	n.d.	[40]
Chlorella sp.	Methanol	7.92 g/L (1 v/v %)	45 days	91% stimulation	40% increase in lipid content	[41]
Chlorella sorokiniana	Methanol	0.5 g/L (500 ppm)	10 days	69% increase	160% increase in Chl a productivity	[43]
Scenedesmus obliquus	Methanol	3.96 g/L (0.5 v/v %)	120 h	133% stimulation	20% decrease in LHCII amount[24h]	[44]
Arthrospira platensis	Ethanol	0.15-1.21 g/L	8 days	24% stimulation	n.d.	[55]
Monodus subterraneus	Ethanol	7.89-15.78 g/L (1-2 v/v %)	6 days	13-44% inhibition	n.d.	[70]
Scenedesmus obliquus	Ethanol	1.84 g/L	9 days	3-fold stimulation	n.d.	[56]
Chlorella	Ethanol	1.38 g/L (0.03 M/L)	24 days	140% stimulation[L] 332% increase[H]	n.d.	[63]
Spirulina platensis	Ethanol	16.56 g/L (0.36 M)	8 days	50 % inhibition	50 % inhibition of oxygen evolution	[68]

					rate at 73 g/L (1.59 M)	
Synechocystis sp.	Ethanol	11.83 g/L<br>(1.5 v/v %)	24h	50 %<br>inhibition	Cell aggregation<br>Chlorophyll a<br>(100% increase)	[69]
Synechocystis sp.	Ethanol<br>Butanol<br>Hexane	2 g/L	20h	No effect<br>48% inhibition<br>54% inhibition	n.d.	[71]
Synechococcus elongatus	Ethanol<br>Butanol<br>Hexane	2 g/L	20h	No effect<br>40% inhibition<br>91% inhibition		
Chlorella vulgaris<br>(0.5 v/v %)	Ethanol<br>Methanol<br>DMSO<br>DMF	3.94 g/L<br>3.96 g/L<br>5.5 g/L<br>4.72 g/L	4 days	86% inhibition<br>69% inhibition<br>No inhibition<br>7% inhibition	n.d.	[46]
Selenastrum capricornutum<br>(0.5 v/v %)	Ethanol<br>Methanol<br>DMSO<br>DMF	3.94 g/L<br>3.96 g/L<br>5.5 g/L<br>4.72 g/L		37% inhibition<br>21% inhibition<br>13% inhibition<br>38% inhibition		
Euglena gracilis	Ethanol	4.6 g/L<br>(100 mM)	20 days	200%<br>stimulation	β-carotene (102% increase)<br>Chlorophyll (98% increase)<br>α-Tocopherol (7-fold decrease)	[53]
Euglena gracilis	Ethanol	10 g/L	7 days	57%<br>Decrease[G]	Vitamin A (105% increase)[G]<br>Vitamin E (105% increase)[G]	[54]
Euglena gracilis (wild)	Ethanol	10 g/L	72h	163%<br>stimulation	α-Tocopherol<br>(39% increase)	[52]
Euglena gracilis (chloroplast-deficient)				142%<br>stimulation	α-Tocopherol<br>(62% increase)	
Scenedesmus sp.	Ethanol	1.42 g/L<br>(0.18 v/v %)	9 days	50%<br>stimulation	n.d.	[57]
Scenedesmus sp.	Ethanol	1.42 g/L<br>(0.18 v/v %)	10 days	9.8-fold<br>stimulation	34 % increase in lipid content	[58]
				3-fold<br>stimulation	24% decrease in lipid content	
Nannochloropsis sp.	Ethanol	1.38 g/L<br>(30 mM)	7 or 8 days	1.3-fold<br>stimulation<br>(Mix)	4-fold increase in respiratory rate<br>Increase in C16:0<br>Decrease in C18:1	[59]
				32% decrease<br>(Het)	3.4-fold increase in respiratory rate<br>Increase in C16:0, C18:0<br>Decrease in C18:1, C20:5	

Chlorella kessleri	Ethanol	2.3 g/L (50 mM)	3 weeks	2.5-fold stimulation	Increase in C16:0 Decrease in C16:1, C16:2	[60]
Dunaliella tertiolecta	Methanol	23 g/L (23000 ppm)	96h	50% Inhibition	n.d.	
	Ethanol	16 g/L (16000 ppm)				
	DMSO	21 g/L (21000 ppm)				
	DMF	15 g/L (15000 ppm)				
	Acetone	10 g/L (10000 ppm)				
Isochrysis galbana	Methanol	21 g/L (21000 ppm)	96h	50% Inhibition	n.d.	[49]
	Ethanol	15 g/L (15000 ppm)				
	DMSO	5 g/L (5000 ppm)				
	DMF	7 g/L (7000 ppm)				
	Acetone	4 g/L (4000 ppm)				
Heterosigma akashiwo	Methanol	0.5 g/L (500 ppm)	96h	50% Inhibition	n.d.	
	Ethanol	2.5 g/L (2500 ppm)				
	DMSO	7 g/L (7000 ppm)				
	DMF	7 g/L (7000 ppm)				
	Acetone	3 g/L (3000 ppm)				
Chlorella pyrenoidosa	Acetone	12 g/L (1.52 v/v %)	96h	50% Inhibition	n.d.	[48]
	Ethanol	1.42 g/L (0.18 v/v %)				
	Methanol	6.33 g/L (0.8 v/v %)				
	DMSO	16.39 (1.49 v/v %)				
	DMF	9.44 g/L (1 v/v %)				
Pseudokirchneriella subcapitata	Acetone	6.4 g/L	72h	50% Inhibition	n.d.	[75]
Pseudokirchneriella subcapitata	Acetone	5.28 g/L	48h	50% Inhibition	n.d.	[74]
Pseudokirchneriella subcapitata	Acetaldehyde	0.017 mg/L	48h	50% Inhibition	n.d.	[74]
Pseudokirchneriella subcapitata	Butanone	8.6 g/L	72h	50% Inhibition	n.d.	[76]
Pseudokirchneriella subcapitata	Butanol<br>Isobutanol	1.56 g/L<br>1.69 g/L	72h	50% Inhibition	n.d.	[75]
Anabaena variabilis	Hexane	43.75 g/L (6.58 v/v %)	10-14 days	50% inhibition	n.d.	[80]
	DMSO	39.27 g/L (3.57 v/v %)				
Anabaena inaequalis	Hexane	11.13 g/L (1.7 v/v %)				
	DMSO	18.8 g/L (1.71 v/v %)				
	Decanol<br>Octanol	2.1 mg/L<br>27.7 mg/L	72h	50%	n.d.	[75]

Pseudokirchneriella subcapitata	Hexanol<br>Pentanol<br>Butanol	115 mg/L<br>370 mg/L<br>1561 mg/L		Inhibition		
Pseudokirchneriella subcapitata	1-propanol<br>2-propanol	4.95 g/L<br>8.47 g/L	48h	50% Inhibition	n.d.	[74]
Chlorella vulgaris	Isopropanol (IPA)	16 g/L	360 h	47% inhibition	IPA conversion to acetone	[81]
Pseudokirchneriella subcapitata	1-butanol<br>Iso-butanol	1.56 g/L<br>1.69 g/L	72h	50% Inhibition	n.d.	[75]
			Glycols			
Selenastrum capricornutum	EG<br>PG	10.9 g/L<br>20.6 g/L	96 h	50% Inhibition	n.d.	[83]
Pseudokirchneriella subcapitata	EG<br>EGBE	36.6 g/L<br>1.84 g/L	72h	50% Inhibition	n.d.	[75]
Pseudokirchneriella subcapitata	EGBE	1.84 g/L	72h	50% Inhibition	n.d.	[84]
Chlorella protothecoides	EG<br>PG	2.59 g/L<br>2.1 g/L	10 days	Growth confirmed	Acidification of medium	[85]
			Cyclic solvents			
Chlorella pyrenoidosa	Furanidine (THF)	2.57 g/L<br>(0.29 v/v %)	96h	50% Inhibition	n.d.	[48]
Scenedesmus quadricauda	Dioxane	5.6 g/L	8 days	Toxicity threshold	n.d.	[86]
Microcystis aeruginosa	Dioxane	0.575 g/L	8 days	Toxicity threshold	n.d.	[86]
Pseudokirchneriella subcapitata	Cyclohexane<br>Cyclohexanol<br>Cyclohexanone	19.3 mg/L<br>411 mg/L<br>1.16 g/L	72h	50% Inhibition	n.d.	[75]
Chlorella	Cyclohexane	1.558 g/L<br>(0.2 v/v %)	10 days	Full growth inhibition	n.d.	[63]
Chlorella	Cyclohexane	1.558 g/L<br>(0.2 v/v %)	25 days	100-150% stimulation	n.d.	[63]
			Chlorinated solvents			
Chlamydomonas reinhardtii	Trichloromethane	13.3 mg/L	72h	50% inhibition	n.d.	[90]
Chlorella vulgaris	DCM<br>Trichloroethylene	2 μg/L-2 mg/L<br>3 μg/L-3 mg/L	8 days	No effect on growth	n.d.	[89]
Selenastrum capricornutum	DCM<br>Trichloroethylene	2 μg/L-2 mg/L<br>3 μg/L-3 mg/L	8 days	No effect on growth	n.d.	[89]
Volvulina steinii	DCM<br>Trichloroethylene	2 μg/L-2 mg/L<br>3 μg/L-3 mg/L	8 days	100% inhibition and cell death	n.d.	[89]
Raphidocelis subcapitata	Trichloroethylene (glass enclosure assay)	0.55 g/L<br>0.1 g/L	72h<br>72h	50% inhibition<br>23% stimulation	n.d.	[92]
Raphidocelis subcapitata	Trichloroethylene (plate assay)	0.45 g/L<br>0.05 g/L	144h<br>144h	50% inhibition<br>72% stimulation	n.d.	[92]
Desmodesmus subspicatus	Trichloroethylene (glass enclosure assay)	0.3 g/L	72h	50% inhibition	n.d.	[92]
Desmodesmus subspicatus	Trichloroethylene (plate assay)	0.35 g/L	72h	50% inhibition	n.d.	[92]
Chlorella kessleri	Trichloroethylene (glass enclosure assay)	0.5 g/L	24h	50% inhibition	n.d.	[92]
Chlorella kessleri	Trichloroethylene (plate assay)	0.2 g/L	24h	50% inhibition	n.d.	[92]

Chlamydomonas reinhardtii	Trichloroethylene<br>Tetrachloroethylene	36.5 mg/L<br>3.64 mg/L	72h	50% inhibition	n.d.	[90]
Synechococcus elongatus	Trichloroethylene<br>Tetrachloroethylene<br>Tetrachloroethane	1.357 g/L (0.093 v/v %)<br>0.149 g/L (0.0092 v/v %)<br>2.86 g/L (0.18 v/v %)	24h	36% inhibition<br>50% inhibition<br>59% inhibition	Increase in lipid peroxidation and activity of SOD and Peroxidase Decrease in Chl content/cell	[91]
Chlamydomonas reinhardtii	Tetra-chloromethane	0.246 mg/L	72h	50% inhibition	n.d.	[90]
Pseudokirchneriella subcapitata	Chloroform<br>Tetra-chloromethane	233 mg/L<br>10.7 mg/L	72h	50% Inhibition	n.d.	[75]
Pseudokirchneriella subcapitata	*trans*-1,2-dichloroethylene<br>*cis*-1,2-dichloroethylene	36.4 mg/L<br>59.7 mg/L	48h	50% inhibition	n.d.	[74]
			Aromatic solvents			
Amphidinium carterae	Benzene<br>Toluene<br>Xylene	0.1-10 mg/L	2nd or 3rd day of logarithmic growth	35% inhibition<br>30% inhibition<br>15% stimulation	n.d.	[96]
Skeletonema costatum	Benzene<br>Toluene<br>Xylene	0.1-10 mg/L	2nd or 3rd day of logarithmic growth	No effect<br>No effect<br>25%-0% inhibition	n.d.	
Dunaliella tertiolecta	Benzene<br>Xylene<br>Toluene	0.1-10 mg/L	2nd or 3rd day of logarithmic growth	10% stimulation<br>20% stimulation to 10% inhibition	n.d.	
Cricosphaera carterae	Benzene<br>Toluene<br>Xylene	0.1-10 mg/L	2nd or 3rd day of logarithmic growth	No effect<br>35% stimulation<br>20% stimulation	n.d.	
Pseudokirchneriella subcapitata	Benzene<br>Toluene<br>Nitrobenzene	15.7 mg/L<br>14.2 mg/L<br>13.9 mg/L	48h	50% Inhibition	n.d.	[74]
Pseudokirchneriella subcapitata	Benzene<br>Toluene<br>Xylene	124 mg/L<br>25.5 mg/L<br>8-26 mg/L	72h	50% Inhibition	n.d.	[75]
Selenastrum capricornutum	BTEX (52% benzene, 28% toluene, 5% ethylbenzene, 5% of *o*-, *m*- and *p*-xylene)	22.7 mg/L	8 days	50% inhibition	Possible damage to membrane integrity	[101]
Scenedesmus obliquus	*m*-Cresol<br>0.162 g/L (1.5 mM)	1.5 mM (CO_2)<br>1.5 mM (glc)<br>1.5 mM (CO_2+glc)<br>1.5 mM (limCO_2)	5 days	No effect<br>81% stimulation<br>10% inhibition<br>47% stimulation	No stress effect on photosynthetic apparatus observed	[109]
Ochromonas danica	*p*-Cresol	0.054-0.432 g/L (0.5-4 mM)	up to 12 days	Growth supported in the dark	n.d.	[107]
Scenedesmus obliquus	*p*-Cresol	0.016 g/L (0.15 mM)	5 days<br>1 day	20% stimulation<br>No effect	No stress effect on photosynthetic apparatus	[108]

Microcystis aeruginosa	Benzene	50-100 μg/L	4 days	No change	No change in microcystin content	[99]
Microcystis aeruginosa	Nitrobenzene	200 μg/L	5 days	10% inhibition	48% increase in protein productivity	[103]
Microcystis aeruginosa	Nitrobenzene	138-294 μg/L different initial cell densities	120h	50% Inhibition	34% decrease in intracellular microcystin-LR productivity	[102]
Skeletonema costatum *Selenastrum capricornutum*	Ethylbenzene	7.7 mg/l<br>3.6 mg/l	96h	50% Lethal effect	n.d.	[100]
Pseudokirchneriella subcapitata	Ethylbenzene	1.34 mg/L	48h	50% Inhibition	n.d.	[74]
Pseudokirchneriella subcapitata	Benzonitrile	23 mg/L	48h	50% Inhibition	n.d.	[74]
Pseudokirchneriella subcapitata	Benzonitrile	121–142 mg/L	48h	50% Inhibition	n.d.	[104]
Chlorella vulgaris	Pyridine<br>α-picoline<br>β-picoline	1 g/L 0.102 v/v %<br>1.05 g/L 0.112 v/v %<br>0.88 g/L 0.094 v/v %	14 days	50% Inhibition	n.d.	[105]
			Chlorinated Aromatic solvents			
Pseudokirchneriella subcapitata	Chlorobenzene<br>1,2-dichlorobenzene,<br>1,2,4-trichlorobenzene<br>1,3,5-trichlorobenzene	7.8 mg/L<br>2.85 mg/L<br>0.64 mg/L<br>1.68 mg/L	48h	50% Inhibition	n.d.	[74]
Cyclotella meneghiniana	1,2,4-Trichlorobenzene	0.245 mg/L (0.245 ppm)	5 days	n.d.	Increase in chloroplast lipids, mitochondria, vacuole (autophagic, central), C16:0, C18:0, C18:1, C20:5. Decrease in nucleus, lipids, vacuole (fibrous), C14:0, C16:1	[94]

PA – photosynthetic activity
A – if compared to autotrophic growth
L – growth in the presence of light
H – heterotrophic growth
G – if compared to glucose based growth

Table S2. Effect of ionic liquids (ILs) and cultivation parameters on microalgae growth and metabolism.

**Strain**	**ILs**	**Conc.**	**Exposure time**	**Effect on growth**	**Effect on metabolism**	**Ref.**
Pseudokirchneriella subcapitata	$[C_3MIM]Br$<br>$[C_3MPy]Br$	>205 g/L (>1000 mM)<br>11.59 g/L (53.7 mM)	up to 2 h	50% inhibition[PA]	Decreased oxygen evolution rate	[110]
Scenedesmus rubescens	$[C_4MIM]BF_4$	>200 mg/L<br>>200 mg/L	24h<br>72h	50% inhibition	n.d.	[114]
	$[C_8MIM]BF_4$	2.97 mg/L	24h			

		0.31 mg/L	72h			
Scenedesmus obliquus	[C$_4$MIM]Br	40 mg/L	24h	50% inhibition	n.d.	[115]
		24.1 mg/L	48h			
		23.6 mg/L	72h			
		22.2 mg/L	96h			
	[C$_6$MIM]Br	17.67 mg/L	24h			
		14.7 mg/L	48h			
		8.63 mg/L	72h			
		5.88 mg/L	96h			
Chlorella ellipsoidea	[C$_4$MIM]Br	26.95 mg/L[25T]<br>24.2 mg/L[28T]	96h	50% inhibition		
	[C$_6$MIM]Br	12.59 mg/L[25T]<br>10.83 mg/L[28T]				
Pseudokirchneriella subcapitata	[C$_4$MPy]Br	1.127 g/L (4.9 mM)	96h	50% inhibition	n.d.	[111]
	[C$_8$MPy]Br	5.72 mg/L (20 μM)				
	[C$_4$MPyrr]Br	2.73 g/L (12.3 mM)				
	[C$_8$MPyrr]Br	13.3 mg/L (48 μM)				
Pseudokirchneriella subcapitata	[C$_4$Py]Tf$_2$N	7.05 mg/L	72h	50% inhibition	n.d.	[112]
	[C$_4$MPyr]Tf$_2$N	>100 mg/L				
	[C$_4$MIM]Tf$_2$N	26.5 mg/L				
Selenastrum capricornutum	[C$_4$MIM]Br	0.466 g/L (2.13 mM)	96h	50% inhibition	n.d.	[119]
	[C$_4$MIM]Cl	0.5 g/L (2.88 mM)				
	[C$_4$MIM]BF$_4$	0.567 g/L (2.51 mM)				
	[C$_4$MIM]PF$_6$	0.372 g/L (1.31 mM)				
	[C$_4$MIM]SbF$_6$	0.05 g/L (0.135 mM)				
Raphidocelis subcapitata	[C$_4$MPyr]BF$_4$	353 mg/L	72h	50% inhibition	n.d.	[47]
	[N$_{4,4,4,4}$]BF$_4$	17.2 mg/L				
	[(Hex)$_3$(TDec)P]Cl	0.084 mg/L				
Scenedesmus obliquus	[C$_8$MIM]Cl	1.36 mg/L	48h	50% inhibition	Damage to cell wall and membranes.<br>Damaged structures of chloroplasts, thylakoids and mitochondria.<br>Increased deposits in vacuoles.	[125]
	[C$_{12}$MIM]Cl	0.027 mg/L				
	[C$_{16}$MIM]Cl	0.012 mg/L				
Selenastrum capricornutum	[C$_4$MIM]Cl	38.5 mg/L	48h	50% inhibition	n.d.	[116]
	[C$_{12}$MIM]Cl	1.1 μg/L				
	[C$_{16}$MIM]Cl	4.1 μg/L				
	[C$_{18}$MIM]Cl	12.9 μg/L				
Scenedesmus quadricauda	[C$_4$MIM]Cl	17.46 mg/L (0.1 mM)	15 days	~50% inhibition	Inhibition of esterase activity.<br>Inhibition of chlorophyll fluorescence.	[133]
Dunaliella tertiolecta	[C$_4$MIM]BF$_4$	100 mg/L	24h	16% inhibition[30]	Carotenoid increase (75%)<br>Chlorophyll increase (500%)	[113]
				48% inhibition[35]	Carotenoid increase (25%)<br>Chlorophyll increase (160%)	
	[C$_8$MIM]BF$_4$	100 mg/L	24h	58% inhibition[30]	Carotenoid increase (50%)	

					Chlorophyll increase (466%)	
				48% inhibition^{35}	Carotenoid increase (225%)<br>Chlorophyll increase (233%)	
Skeletonema marinoi	[C$_4$MIM]Cl	21 mg/L<br>(0.12 mM)	72h	50% inhibition	Interference in silica uptake and cell wall organization$^{(0.1\text{-}0.3\&1.9)}$	[128]
Phaeodactylum tricornutum		(220 mg/L)<br>1.26 mM	72h	50% inhibition		
Synechococcus sp.	[HOC$_2$MIM]Cl	120 mg/L	96h	No effect on growth	Increase in soluble protein content (136%)<br>Increase in POD activity (110%), SOD activity (33%) and CAT activity (75%)<br>Increase in MDA content (145%)	[130]
Phaeodactylum tricornutum	[C$_8$MIM]Br	8.9 mg/L	96h	50% inhibition	No change in Chl *a* content$^{10mg/L}$<br>Increase in soluble protein content (60%)$^{10mg/L}$<br>Increase in SOD activity (44%)$^{10mg/L}$<br>Increase in MDA content (~60%)$^{10mg/L}$	[131]
Skeletonema costatum	[C$_8$MIM]Br	40 mg/L	96h	50% inhibition	Decrease in Chl *a* content (43.8%)<br>Increase in soluble protein content (100%)<br>Increase in SOD activity (84%)<br>Increase in ROS level (316%) and MDA content (163%)	[132]
Raphidocelis subcapitata	[MOC$_2$MPyr]NTf$_2$	(0.55 g/L)<br>1.3 mM	72h	50% inhibition	n.d.	[122]
		(0.38 g/L)<br>0.9 mM	72h	Limited inhibition	Increase in protein content (32%)	
	[MOC$_2$MPyr]BF$_4$	(0.55 g/L)<br>2.4 mM	72h	50% inhibition	n.d.	
		(0.39 g/L)<br>1.7 mM	72h	Limited inhibition	Increase in protein content (22%)	
Scenedesmus obliquus	L-(+)-[C$_2$MIM]L	>1 g/L<br>(>5 mM)	24h	50% inhibition	Increase in ROS production (22%)^{5mM}	[123]
	D-(-)-[C$_2$MIM]L	0.45 g/L<br>(2.25 mM)			Increase in ROS production (233%)^{5mM}	
Euglena gracilis	L-(+)-[C$_2$MIM]L	1.31 g/L<br>(6.58 mM)				
	D-(-)-[C$_2$MIM]L	1.25 g/L<br>(6.24 mM)			n.d.	
Scenedesmus obliquus	L-(+)-[HMIM]T	16 mg/L	24h	50% inhibition	Increase in CMP (530%)$^{15mg/L}$	[124]
		7.9 mg/L	48h		Increase in CMP (150%)$^{10mg/L}$	
	D-(-)-[HMIM]T	28.3 mg/L	24h		Increase in CMP (479%)$^{25mg/L}$	
		12.2 mg/L	48h		Increase in CMP (120%)$^{10mg/L}$	
	[OHC$_2$MIM]I	>0.254 g/L<br>(>1 mM)				

Scenedesmus vacuolatus	[OHC$_2$MIM]NTf$_2$	61 mg/L (150 μM)	24h	50% inhibition	n.d.	[120]
	[C$_2$MIM]Cl	88.2 mg/L (602 μM)				
	[C$_8$MIM]Cl	0.46 μg/L (0.002 μM)				
	[C$_{10}$MIM]Cl	0.077 μg/L (0.3 nM)				
Scenedesmus vacuolatus	[MPhBIM]Br	10.33 μg/L (0.035 μM)	24h	50% inhibition	n.d.	[121]
	[C$_2$OPhBIM]Br	13.66 μg/L (0.042 μM)				
	[C$_2$PhBIM]Br	0.513 mg/L (1.66 μM)				
	[C$_2$PhBIM]I	0.345 mg/L (0.97 μM)				
Chlorella vulgaris	[MDPh(Py)AcOM]Br	441 mg/L	72h	50% inhibition	n.d.	[117]
	[MDPh(PyAcO)AcOM]Br	294 mg/L				
Pseudokirchneriella subcapitata	[MDPh(Py)AcOM]Br	587 mg/L				
	[MDPh(PyAcO)AcOM]Br	281 mg/L				
Raphidocelis subcapitata	[Chol]Bic	232 mg/L	72h	50% inhibition	n.d.	[118]
	[Chol]Bit	27 mg/L				
	[Chol]DHCit	87 mg/L				
	[Chol]Cl	72 mg/L				
	[Bzchol]Cl	196 mg/L				

CMP – cell membrane permeability
NTf$_2$=N(CF$_3$SO$_2$)$_2$

S3. Calculation scheme

1. Calculation Procedure

Fundamental energy requirements and production cost were analysed for isolation of demanded product. The analyses were carried out in simplified form under following assumptions: 1) total solvent recovery, 2) no heat losses, 3) no heat recovery and 4) equipment amortization is not taken into account.

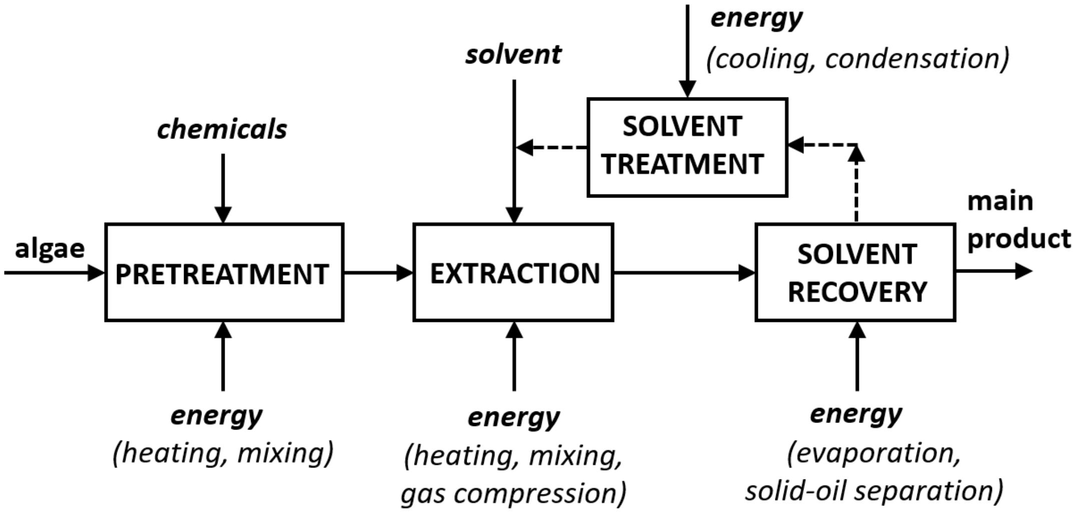

Figure S1. Scheme of Calculation Procedure.

Figure S1. shows a model for the calculation procedure. All lab-scale technologies are composed of these technological steps - pretreatment, extraction and solvent recovery including its recycling. The specific energy requirement E_{SEP} (J kg^{-1}) and the specific production cost C_{SEP} (€ kg^{-1}) of separation process used were calculated as follows:

$$E_{SEP} = E_{TOTAL} / m_{PRODUCT} \tag{1}$$

$$C_{SEP} = C_{TOTAL} / m_{PRODUCT} \tag{2}$$

where E_{TOTAL} is total energy requirement of separation process (J), C_{TOTAL} is total costs for product separation (€) and $m_{PRODUCT}$ is weight of the product (kg) defined as

$$m_{product} = w_{dB} \cdot m_{wB} / y_{product} \tag{3}$$

where m_{wB} is the mass of wet biomass (kg), w_{dB} is mass fraction of dried biomass (-) and $y_{product}$ is the yield of product related to dried biomass (-).

The total energy demand of extraction using liquid solvent was calculated:

$$E_{TOTAL} = E_{PT} + E_{EM} + E_{SSP} + E_{SC} \tag{4}$$

where E_{PT} is the energy needed for pretreatment (J), E_{EM} is the energy needed for mixing during extraction (J), E_{SSP} is the energy needed for solvent separation from an extract (J) and E_{SC} is the energy needed for reverse solvent condensation (J).

The energy requirement needed for pretreatment E_{PT} was calculated:

$$E_{PT} = P_{PT} \cdot t_{PT} = \varepsilon_{PT} \cdot V_{PT} \cdot t_{PT} \tag{5}$$

where P_{PT} is the power input of equipment used for pretreatment (W), V_{PT} is the volume of pretreated mixture (m^3), t_{PT} is the time of pretreatment (s) and ε_{PT} is the specific power requirement of pretreatment (W m^{-3}).

The energy requirement needed for mixing during extraction was calculated:

$$E_{EM} = \varepsilon_{EM} \cdot V_{EM} \cdot t_{EM} \tag{6}$$

where ε_{EM} is the specific power input for mixing (W m^{-3}), V_{EM} is the volume of mixture during extraction (m^3), t_{EM} is the time of mixing during extraction (s). The specific power input for mixing ε_{EM} = 300 W m^{-3} was assumed for calculation.

Assuming that the multi-component solvent is totally separated from an extract by the evaporation the energy needed for separation was calculated in simplified form as follows:

$$E_{SSP} = \sum_j \Delta H_j^{vap}(T) \cdot m_{S-j} \tag{7}$$

where ΔH^{vap}_j (T) is the heat of vaporization of jth component of the solvent solution (J kg^{-1}) at temperature T (K) and m_{S-j} is the mass of jth component of the solvent solution (kg). The heat of vaporization was calculated using following formula:

$$\Delta H^{vap}(T) = A.\exp(-\alpha \cdot T_r) \cdot (1 - T_r)^{\beta} \tag{8}$$

where A, α and β are parameters overtaken from NIST database for given component, T_r is reduced temperature calculated as ratio of temperature T and critical temperature T_c of given component. The evaporation at normal pressure was assumed. The heats of vaporization were calculated at normal boiling temperature for given component. Assuming that the reverse condensation of solvent components occurs at the same conditions as evaporation the energy needed for condensation E_{SC} equals to E_{SSP}.

The total cost for extraction process was calculated:

$$C_{TOTAL} = C_{CH} + C_{PT} + C_{EM} + C_{SSP} + C_{SC} \tag{9}$$

where C_{CH} is the cost of chemicals (€), C_{PT} is the price of electricity required for pretreatment (€), C_{EM} is the price of electricity required for mixing during extraction (€), C_{SSP} is the price of water steam needed for solvent evaporation (€) and C_{SC} is the price of cooling water needed for reverse solvent condensation (€).

The prices of electricity needed for pretreatment and for mixing during extraction were calculated as follows:

$$C_{PT} = c_{el} \cdot E_{PT} \tag{10}$$

$$C_{EM} = c_{el} \cdot E_{EM} \tag{11}$$

where c_{el} is the price of electricity (€ MJ^{-1}).

The condensation of saturated water steam was assumed as an energy source for solvent evaporation. The price of water steam needed was calculated:

$$C_{SSP} = c_{steam} \cdot (E_{SSP} / \Delta H_{steam}^{cond}) \tag{12}$$

where c_{steam} is the price of water steam (€ kg^{-1}) and ΔH^{cond}_{steam} (T_{cond}) is the heat of condensation of water steam at condensation temperature T_{cond}. The saturated water steam at temperature of 150°C was assumed for solvent evaporation.
The price of cooling water needed for solvent condensation was calculated:

$$C_{SC} = c_{cw} \cdot (E_{SC} /(c_{p_{cw}} \cdot \Delta T_{cw})) \tag{13}$$

where c_{cw} is the price of cooling water (€ kg^{-1}), c_{pcw} is the specific heat capacity of cooling water (J $kg^{-1}K^{-1}$) and ΔT_{cw} is allowed temperature increase of cooling water. The allowed temperature increase of 15 K and specific heat capacity of cooling water of 4 182 J $kg^{-1}K^{-1}$ were assumed and used for calculation.

The costs of the chemicals were estimated on the basis of the following prices: 1) chloroform p.a.: 5 750 € m^{-3}, 2) hexane p.a.: 20 500 € m^{-3}, 3) dichloromethane p.a.: 6 800 € m^{-3}, 4) methanol p.a.: 2 300 € m^{-3}, 5) acetone p.a.: 2 900 € m^{-3}, 6) ethyl acetate p.a.: 93 000 € m^{-3}, 7) ionic liquid THPC: 271 000 € m^{-3}, 8) ionic liquid [BMIM]HSO4: 590 500 € m^{-3}, 9) ionic liquid EMIM DBP: 135 000 € m^{-3}, 10) water: 4 € m^{-3}, 11) CO2 (food quality): 1.8 € kg^{-1} and 12) ethanol absolute: 28.5 € kg^{-1}.
The energy costs were estimated on the basis of the actual mean prices: 1) electricity: 126 000 € MJ^{-1}, 2) saturated water steam: 20 € t^{-1}, 3) cooling water: 0.1 € t^{-1}.

The error of presented estimations is 20 % in maximum for both energy requirement and production costs.

2. Supercritical Extraction Technology

The supercritical extraction was calculated under following assumptions: 1) two-stage solvent compression with inter- and after cooling of compressed solvent, 2) reversible adiabatic compression, 3) adiabatic efficiency of 60 % for irreversible compression, 4) mechanical efficiency of 96 % of driving unit, 5) inlet temperature of 20°C and pressure of 101.325 kPa of the solvent before first-stage compression, 6) outlet solvent temperature from coolers equals to extraction temperature reported in the cited article and 7) Poisson constant κ = 1.29.

The total energy requirement of supercritical extraction was calculated as

$$E_{total} = E_C + E_{GSC} \tag{14}$$

where E_C is the energy needed for solvent compression (J) and E_{GSC} is the energy needed for cooling of compressed solvent cooling after compression (J).

The energy needed for solvent compression in i^{th} compression stage was calculated as follows:

$$E_{Ci} = n_{solvent} \cdot (1/\eta_{ad}) \cdot (1/\eta_m) \cdot w_{t-rev} \tag{15}$$

where

$$w_{t-rev} = (\kappa /(1-\kappa)) \cdot p_{in} \cdot v_{in} \cdot \left\lfloor (p_{in} / p_{out})^{(1-\kappa)/\kappa} - 1 \right\rfloor \tag{16}$$

where $n_{solvent}$ is the number of moles of compressed solvent (mol), p_{in} is the stage inlet pressure (Pa), p_{out} is the stage outlet pressure (Pa), v_{in} is molar volume of the solvent in the stage inlet (m^3 mol^{-1}), η_{ad} is the adiabatic efficiency of irreversible compression (-), η_m is the efficiency of the driving unit (-), w_{t-rev} is the shaft work of reversible compression (J mol^{-1}) in the stage and κ is the Poisson constant (-).

The pressure between compression stages was estimated using formula:

$$p_{12} = (p_{in-1} \cdot p_{out-2})^{1/2} \tag{17}$$

where p_{in-1} is the inlet pressure to the compressor, p_{out-2} is the outlet pressure from the compressor.

The energy needed for cooling of compressed solvent after i^{th} compression stage was calculated as follows:

$$E_{GSCi} = n_{solvent} \cdot \sum_j x_j \cdot (-\Delta h_j^{cooling}) \quad (18)$$

where

$$\Delta h_j^{cooling} = \int_{Tin-c}^{Tout-c} c_{p_j}(T) \cdot dT \quad (19)$$

where x_j is the mole fraction of jth solvent component (-), $\Delta h^{cooling}{}_j$ is the enthalpy change of jth solvent component during solvent cooling (J mol^{-1}), T_{in-c} and T_{out-c} are the temperatures at inlet and outlet of cooler of [ith] compression stage (K) and c_{pj} (T) is the temperature dependence of molar heat capacity of jth solvent component (J mol^{-1}K^{-1}).

The inlet temperature to the cooler T_{in-c} was calculated from the following relation:

$$w_{t-irrev} = w_{t-rev} \cdot (1/\eta_{ad}) = \overline{c_p} \cdot (T_{in-c} - T_{in}) \quad (20)$$

where T_{in} is the solvent temperature at stage inlet (K), $\overline{c_p}$ is the average molar heat capacity of the solvent in given temperature range (J mol^{-1}K^{-1}). It was found that gas behavior in stage output is closed to ideal gas behavior. Therefore, the molar heat capacity for ideal gas was used for calculation in this case.

The total cost for supercritical extraction was calculated:

$$C_{total} = C_C + C_{GSC} \quad (21)$$

where C_C is the price of electricity needed for solvent compression (€) and C_{GSC} is the price of cooling water needed for cooling of compressed solvent after compression (€).

The price of electricity needed for compression was calculated as follows:

$$C_C = c_{el} \cdot E_C \quad (22)$$

where c_{el} is the price of electricity (€ MJ^{-1}). The price of cooling water needed for cooling of compressed solvent after compression was calculated:

$$C_{GSC} = c_{cw} \cdot (E_{GSC} / (c_{p_{cw}} \cdot \Delta T_{cw})) \quad (23)$$

where c_{cw} is the price of cooling water (€ kg^{-1}), c_{pcw} is the specific heat capacity of cooling water (J mol^{-1}K^{-1}) and ΔT_{cw} is allowed temperature increase of cooling water. The allowed temperature increase of 15 K and specific heat capacity of cooling water of 4 182 (J mol^{-1}K^{-1}) were assumed and used for calculation.
